# Self-Referential Processing Effects of Non-invasive Brain Stimulation: A Systematic Review

**DOI:** 10.3389/fnins.2021.671020

**Published:** 2021-06-10

**Authors:** Zhongjie Bao, Belal Howidi, Amer M. Burhan, Paul Frewen

**Affiliations:** ^1^Department of Psychiatry, Schulich School of Medicine and Dentistry, London, ON, Canada; ^2^Interdisciplinary Program in Neuroscience, Western University, London, ON, Canada; ^3^Ontario Shores Centre for Mental Health Sciences, Whitby, ON, Canada; ^4^Department of Psychiatry, Temerty School of Medicine, University of Toronto, Toronto, ON, Canada

**Keywords:** self-referential processing, neuromodulation, non-invasive brain stimulation (NIBS), systematic review, bodily self-consciousness

## Abstract

Systematic reviews of neuroimaging studies confirm stimulus-induced activity in response to verbal and non-verbal self-referential processing (SRP) in cortical midline structures, temporoparietal cortex and insula. Whether SRP can be causally modulated by way of non-invasive brain stimulation (NIBS) has also been investigated in several studies. Here we summarize the NIBS literature including 27 studies of task-based SRP comparing response between verbal and non-verbal SRP tasks. The studies differed in design, experimental tasks and stimulation parameters. Results support the role of left inferior parietal lobule (left IPL) in verbal SRP and for the medial prefrontal cortex when valenced stimuli were used. Further, results support roles for the bilateral parietal lobe (IPL, posterior cingulate cortex), the sensorimotor areas (the primary sensory and motor cortex, the premotor cortex, and the extrastriate body area) and the insula in non-verbal SRP (bodily self-consciousness). We conclude that NIBS may differentially modulate verbal and non-verbal SRP by targeting the corresponding brain areas.

## Introduction

What constitutes our sense of self? This question has intrigued philosophers, psychologists, and neuroscientists alike for centuries. William James (1890) early subject-object framework distinguished the experience of self-referential processing (SRP) into its task vs. stimulus aspects, with the content or stimuli of SRP further categorizable into corporal (physical, somatic, non-verbal) versus non-corporal (spiritual, semantic, verbal) referents, and positive versus negative emotional valences (see also Legrand and Ruby, 2009). The distinction between verbal SRP (V-SRP) and non-verbal SRP (NV-SRP) bears significance in recent research topics (Frewen et al., 2020), including psychopathology (LeMoult et al., 2017; Lin et al., 2018; Yoon et al., 2019), neuroendocrinology (Li et al., 2018; van Buuren et al., 2020), and meditation (Katyal et al., 2020).

Researchers have also taken interest in the neurobiological basis of V-SRP and NV-SRP, with neuroimaging literature also providing a basis for distinguishing SRP into verbal (V-SRP) versus non-verbal (NV-SRP) domains (Frewen et al., 2020). Neuroimaging reviews suggested that SRP in general may be associated with activities in the default mode network (DMN) and its sub-systems. Within the DMN, the dorsomedial prefrontal cortex (DMPFC) subsystem consists of the DMPFC, inferior parietal lobule (IPL), the lateral temporal cortex, and the temporal poles, whereas the medial temporal lobe (MTL) subsystem consists of the ventromedial prefrontal cortex (VMPFC), posterior IPL, the retrosplenial cortex and the hippocampus, and the midline core subsystem can be considered as the convergence of parts of the DMPFC and MTL subsystems (Andrews-Hanna et al., 2010; Wen et al., 2020). However, neuroimaging findings in response to SRP tasks further differentiate response among these ROIs. For example, V-SRP is known to be at least partially mediated by DMN activity in the medial prefrontal cortex (MPFC), posterior cingulate cortex (PCC), ventral precuneus, and the bilateral IPL (e.g., Araujo et al., 2015; Davey et al., 2016), although the response to different kinds of meditation practices suggest that it may be particularly the left more so than the right IPL that is associated with V-SRP (e.g., Fingelkurts et al., 2016; Fingelkurts et al., 2020). In contrast, NV-SRP emanating from the inner body (i.e., interoception; e.g., heartbeat) or the outer body (i.e., exteroception; e.g., touch) is assessed during tasks that engage attention toward bodily self-consciousness (BSC) (reviewed by Park and Blanke, 2019). Although interoception is typically associated with activity in the insula and cingulate cortex, exteroceptive aspects of BSC are typically associated with activity in the premotor cortex (PMC), intraparietal sulcus (IPS), and right IPL activity (Park and Blanke, 2019). Park and Blanke (2019) also suggested the existence of an integrated NV-SRP system centered in the IPS with the involvement of the PCC, IPL, PMC, and insula cortex. Further, both VMPFC and DMPFC may be important for valenced self-evaluation (Fingelkurts et al., 2016; Fingelkurts et al., 2020). However, although neuroimaging researchers can draw correlational inferences between SRP and response in various brain regions, causal evidence remains lacking.

One approach to arrive at causal evidence for the involvement of brain regions in SRP would be to modulate the activity of different brain regions and assess the outcomes of doing so for SRP. Emerging literature has therefore also investigated whether subjective and behavioral responses to SRP tasks can be modulated through non-invasive brain stimulation (NIBS) in the form of transcranial magnetic stimulation (TMS) and transcranial direct current stimulation (tDCS). TMS involves stimulating a region of the brain with a powerful magnetic field for a short period using a magnetic coil to induce a current in the cortical neurons parallel to the coil (Hallett, 2000; Barker and Shields, 2017). TMS can be applied phasically using an event-related approach correlated to the presentation of discrete stimuli during an SRP task or repeatedly (rTMS) and tonically over the course of an extended treatment session (e.g., measured in minutes). Typically, single or paired TMS pulses are applied within 500 milliseconds (ms) of stimulus onset during the event-related approach to affect the brain’s response to that stimulus (Miniussi et al., 2013) whereas rTMS applied continuously can be used to affect task performance in general (Beynel et al., 2019), creating “carry-over” effects on neural excitability immediately during and after the stimulation session. As a rule of thumb, low frequency (≤ 1Hz) rTMS reduces cortical excitability whereas high frequency (≥ 5Hz) rTMS increases cortical excitability (Beynel et al., 2019). However, it is important to note that increases or decreases of cortical excitability do not necessarily equate to facilitation or inhibition of certain cognitive functions because the cascade of effects of cortical excitability is modulated by several factors before reaching the level of behavioral impacts (Bestmann et al., 2015).

Whereas TMS induces magnetic fields surrounding the skull to indirectly influence target electrical currents within the brain, tDCS uses a weak (typically ≤ 2.5 mA) direct current constantly applied to either increase or decrease neuronal excitability depending on the polarity. tDCS is almost always used tonically rather than phasically as single pulses to discrete stimuli, due to the weakness in tDCS current strength being unlikely to influence cognition in such fashion. Anodal tDCS is often thought to increase the likelihood of reaching the threshold of the action potential, while cathodal tDCS is thought to inhibit neural activity in the stimulated area (Inukai et al., 2016). However, depending on the distance between the electrodes used in various montages, the electrical field is increased either primarily under cortex positioned between the sites or underneath both sites (Sadleir et al., 2010). Similar to TMS, tDCS does not always yield effects in the desired direction, and “paradoxical” non-linear effects have been described (e.g., Kuo et al., 2013). Moreover, continuous stimulation might influence the mechanism of neurophysiological homeostasis in addition to cortical excitability (Fricke et al., 2011), thus rendering the outcome of the stimulation further uncertain.

With these precautions in mind, a number of NIBS studies show impacts for rTMS and tDCS in cognitive processes and psychopathologies (reviewed by Brunoni and Vanderhasselt, 2014; Dedoncker et al., 2020), suggesting that NIBS might also be used to study SRP. However, NIBS studies on SRP have been relatively scarce. Frewen et al. (2020) briefly overviewed studies whereby NIBS was used to modulate both on-task SRP and spontaneous SRP as it occurs during resting state. Further, Chaieb et al. (2019) systematically reviewed the effects of neuromodulation on mind-wandering which may be considered a form of spontaneous SRP during resting state due to the functional and anatomical overlap between the brain regions mediating mind-wandering and SRP (e.g., Qin and Northoff, 2011). In their review of the tDCS literature, Chaieb and colleagues (2019) identified the dorsolateral prefrontal cortex (DLPFC), ventrolateral prefrontal cortex, the MPFC, and the right IPL as regions involved in mind-wandering, and suggested that tDCS can potentially modulate activity within the MPFC and the right IPL, further suggesting possible applications of NIBS to SRP, although TMS studies were not included. Here, we undertook to what is in our knowledge the first systematic review of the effects of NIBS for on-task SRP that has considered both TMS and tDCS studies and theoretical differentiation between V-SRP and NV-SRP (BSC).

## Methods

**FIGURE 1:**
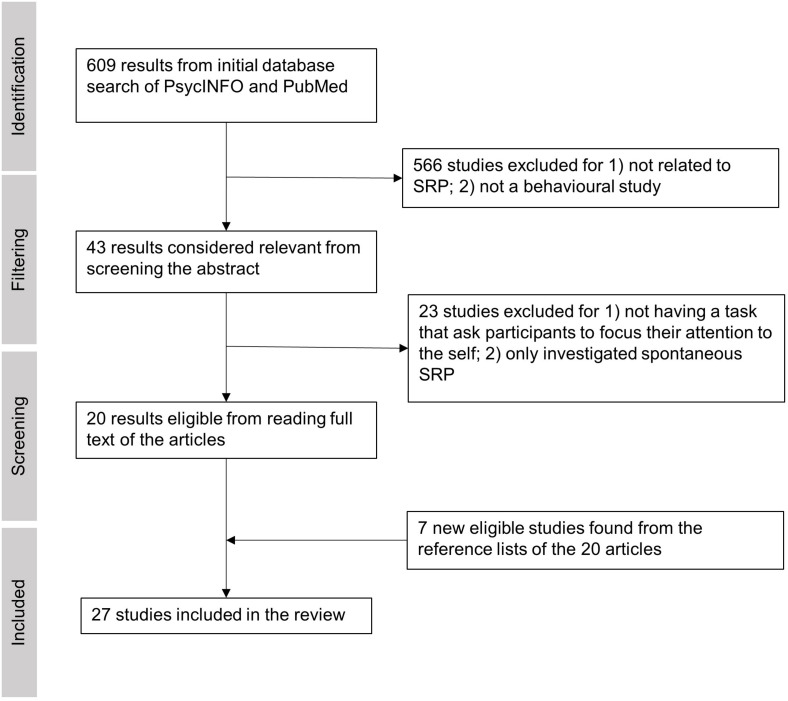
The process of article inclusion and exclusion of the systematic literature review.

We conducted a PsycInfo and PubMed search with the following terms on Apr 13th, 2021: (tDCS OR rTMS OR TMS OR tES) (self refer^∗^ OR self recog^∗^ OR self other OR rubber hand illusion), restricting our search to peer-reviewed journal articles with no restriction on publication time. This search yielded 217 results from PsycInfo and 391 results from PubMed, making a total of 608 results (Figure 1). After an initial screening of each article’s abstract, 43 empirical studies were considered potentially relevant and thus were passed for full-text screening. The screening process and methodological quality evaluation were carried out by two of the authors (ZB and PF) with discussions on each paper. Any uncertainty in agreement on the meeting of inclusion and exclusion criteria were taken up with a third co-author. The 566 excluded articles were either 1) focused on tasks unrelated to SRP or 2) focused on clinical populations or 3) lacked inclusion of a behavioral task. After reading the full texts of the 43 studies, 20 studies qualified for the review because they featured at least one task that required participants to explicitly attend to verbal or non-verbal (bodily) self-referential stimuli (i.e., involved on-task SRP). The 23 excluded studies either: 1) did not include an SRP task condition, or 2) only investigated spontaneous SRP without an explicit task (e.g., SRP occurring in the form of mind wandering during resting state). We decided not to include at-rest SRP studies because this literature was already recently reviewed by Chaieb et al. (2019). For this review, we focus on SRP tasks that required internal attention directed toward oneself in the verbal (V-SRP) or non-verbal sense (NV-SRP) (see Frewen et al., 2020). Comparably, tasks that primarily required attention being directed to other people (e.g., theory of mind tasks) or external stimuli were therefore excluded. Finally, seven new studies from the reference lists of the 20 qualified articles were identified and added to the review, resulting in 27 studies in total (Figure 1). By comparison, the excluded studies are listed in Supplementary Table 1.

From each article we extracted the most relevant experimental variables, that is, the (1) study design (rTMS vs. single-pulse TMS vs. tDCS), (2) NIBS parameters (stimulation site, duration, timing and strength), (3) sample size, (4) SRP task administered, (5) measurement (Tables 1, 2), and (6) findings (Tables 3, 4). We followed the guidelines and used the Cochrane risk-of-bias tool (Higgins et al., 2019) to assess the quality of study methods (Table 5).

**TABLE 1:** Summary of experimental paradigms of V-SRP studies.

Study	Experimental Task	Timing of Task	Design	Sham condition	Site of stimulation	*N(% females)*	Stimulation method, time and intensity	Dependent variable(s)
Schäfer and Frings, 2019	SRET - neutral	Offline	B	No	Anodal/cathodal VMPFC (Fpz), cathodal/anodal DLPFC (F3)	65(72%)	0.5-mA tDCS for 20 min. Target electrodes are 9 cm^2^, reference electrodes are 35 cm^2^	Accuracy and RT
Lou et al., 2004	SRET - neutral	Online	W	No	Oz, Pz, and Fz	25(54%)	Single-pulse TMS at 150% MEP of the feet, at 0∼480 ms post-stimulus	Accuracy and RT
Lou et al., 2010	SRET - neutral	Online	W	No	MPFC, left IPL, right IPL	15(39%)	Single-pulse TMS at 150% RMT, at 0∼480 ms post-stimulus	Accuracy and RT
Barrios et al., 2008	SRET - affective	Online	B	Yes	MPFC, Pz, and SMA	10(100%)	Single-pulse TMS at 90% RMT, 500 ms post-stimulus	Self-enhancement scores and RT
Mainz et al., 2020	SRET - affective	Offline	B	Yes	Anodal/cathodal Fpz (MPFC) and cathodal/anodal Oz (occipital)	75(0%)	2-mA tDCS for 20 min. Target and reference electrodes are 35 cm^2^	Self-enhancement scores
Dedoncker et al., 2019	SRET - affective	Offline	B	Yes	Anodal Left DLPFC, cathodal right supraorbital area	41(100%)	1.5-mA tDCS or 20 mins. Target and reference electrodes are 25 cm^2^	Perceived criticism, current mood, and resting FC
Kwan et al., 2007	SRET - affective	Online	W	Yes	MPFC, Pz, and SMA	12(83%)	Single-pulse TMS at 90% RMT, 500 ms post-stimulus	Self-enhancement scores and RT
Luber et al., 2012	SRET - affective	Online	W	No	MPFC, left IPL, right IPL	18(44%)	Single-pulse TMS at 150% RMT, 0∼480 ms post-stimulus	Self-enhancement scores and RT
De Raedt et al., 2017	SRET - affective	Offline	W	Yes	Anodal Left DLPFC, cathodal right supraorbital area	32(100%)	1.5-mA tDCS for 20 min. Target and reference electrodes are 35 cm^2^	Ruminative thinking, current mood, implicit and explicit self-esteem
De Pisapia et al., 2019	SRET - affective	Offline	W	Yes	MPFC (Fpz)	14(50%)	1-Hz rTMS for 14 min at 100% of RMT	RT and fMRI BOLD signal

*Abbreviations: W, within-subject design; B, between-subject design; RMT, resting motor threshold; RT, reaction time; TMS, transcranial magnetic stimulation; tDCS, transcranial direct current stimulation; rTMS, repetitive transcranial magnetic stimulation; cTBS, Continuous theta-burst stimulation; MPFC, medial prefrontal cortex; VMPFC, ventromedial prefrontal cortex; DLPFC, dorsolateral prefrontal cortex; IPL, inferior parietal lobule; BOLD, blood oxygen level dependant; SMA, supplementary motor area; PPC, posterior parietal cortex; HEP, heartbeat-evoked potential; MEP, motor-evoked potential; EBA, extrastriate body area; aIPS, anterior inferior pareital lobule; M1, primary motor cortex; SRET, self-referential encoding task; RHI, rubber hand illusion; SODT, self-other discrimination task; HBDT, heartbeat detection task.*

**TABLE 2:** Summary of experimental paradigms of NV-SRP studies.

Study	Experimental Task	Timing of Task	Design	Sham condition	Site of stimulation	*N(% females)*	Stimulation method, time and intensity	Type of measure
Payne and Tsakiris, 2017	SODT	Offline	B	Yes	Right IPL (CP6), reference electrode over the Vertex	60(73%)	1-mA tDCS for 20 min. Target and reference electrodes are 3.5 cm^2^	Proportion of morphing video judged “self”
Uddin et al., 2006	SODT	Offline	W	No	Left and Right IPL	8(75%)	1-Hz rTMS for 20 min at 100% RMT	Proportion of morphing pictures judged “self”
Heinisch et al., 2011	SODT	Offline	W	Yes	Left DLPFC (midpoint of triangle F3, F7, Fp1), right DLPFC (midpoint of triangle F4, F8, Fp2), left IPL (CP5), right IPL (CP6)	10(50%)	1-Hz rTMS for 20 min at 100% RMT	Proportion of morphing video judged “self”, self-reported valence of self-recognition
Heinisch et al., 2012	SODT	Offline	W	Yes	Right IPL (CP6)	10(50%)	1-Hz rTMS for 20 min at 100% RMT	Proportion of morphing video that is judged to be the self, self-reported valence of self-recognition
Bassolino et al., 2018	RHI in Virtual reality	Online	Mixed design	No	M1, vertex, 80% RMT for subthreshold stimulation as control	32(50%)	Single-pulse TMS at 130% RMT	PD, MEP, subjective reports of body ownership
Convento et al., 2018	RHI	Online	B	Yes	Right PMC, right IPL	56(95%)	1.5-mA tDCS for 10 min. Target and reference electrodes are 25 cm^2^	PD, subjective reports of body ownership
della Gatta et al., 2016	RHI	Online	B	No	Left M1, right M1 as control	52(64%)	Single-pulse TMS at 110% RMT	PD, MEP, subjective reports of body ownership
Tsakiris et al., 2008	RHI	Online	W	No	Right IPL, vertex	10(60%)	Single-pulse TMS with varying intensity (38-65% maximum stimulator output), 350 ms post-stimulus	PD
Kammers et al., 2009	RHI	Offline	W	Yes	Left IPL (TP3)	13(100%)	1-Hz rTMS for 20 min at 80% RMT	PD, subjective reports of sensations
Wold et al., 2014	RHI	Offline	W	No	Left EBA, 40% RMT stimulation as control	19(58%)	1-Hz rTMS for 20 min at 80% RMT	PD, subjective reports of body ownership
Karabanov et al., 2017	RHI	Online	W	No	Anterior IPS, M1	28(43%)	Single- and paired-pulse TMS at 100% RMT for M1, 90% RMT for aIPS, 500 ms post-stimulus	PD, MEP, subjective reports of body ownership
Fossataro et al., 2018	RHI	Offline	W	Yes	Left M1	48(79%)	1-Hz rTMS for 20 min at 90% RMT and single-pulse TMS at 100% RMT	PD, MEP, subjective reports of body ownership
Hornburger et al., 2019	RHI	Online	W	Yes	Anodal/cathodal S1(C3), reference electrode over right supraorbital region	30(60%)	1-mA tDCS for 20 min. Target and reference electrodes are 35 cm^2^	PD, subjective reports of body ownership
Lira et al., 2018	RHI	Online	W	Yes	Right PPC (35 cm^2^, 2-mA, P4), right PMC (1-mA, fC4, 10-10 EEG system), reference electrode over contralateral supraorbital region (35 cm^2^)	160(71%)	2- or 1-mA tDCS for 10 min. Target and reference electrodes are 35 or 21 cm^2^	PD, subjective reports of body ownership
Peviani et al., 2018	RHI	Offline	W	No	PMC, vertex	24(79%)	1-Hz rTMS for 20 min at 100% RMT	PD, subjective reports of body ownership
Sagliano et al., 2019	HBDT	Offline	W	Yes	Anodal Left insula (midpoint of F7 and T3), cathodal left frontal pole (Fp2); anodal right insula (midpoint of F8 and T4), cathodal right frontal pole (Fp1)	16(56%)	1-mA tDCS for 15 min. Target electrode is 6.25 cm^2^, reference electrode 25 cm^2^	Heartbeat counting accuracy, self-reported state anxiety
Pollatos et al., 2016	HBDT	Offline	W	No	Right insula (FT8), somatosensory cortex (chest location, Cz), central occipital (Oz)	18(0%)	5-Hz cTBS for 40 sec at 80% RMT	Heartbeat and respiratory counting accuracy and confidence in judging accuracy, self-reported state anxiety, HEP

*Abbreviations: see the end of Table 1 for abbreviations.*

**TABLE 3:** Summary of results of the included V-SRP studies.

Study	Task	Main Results	Other Results
**TMS studies**			
Neutral			
Lou et al., 2004	Rate the applicability of personality traits to self, best friend, and the Danish Queen. Then indicate their previous choice as fast as they can	SPE was reduced by TMS to Pz applied 160ms post-stimulus (self > other)	No effect was found in the Fz stimulation condition
Lou et al., 2010	Same as Lou et al., 2004, but without the Danish Queen condition	SPE was reduced by TMS to both left and right IPL applied 160ms, 240ms, and 480 ms post-stimulus. The left IPL had a much stronger effects than right IPL.	No effect was found in the Fz stimulation condition
Affective			
Kwan et al., 2007	Assign positive, neutral and negative adjectives to either the self or their best friend	real stimulation over the MPFC reduced SEB compared to sham	Precuneus stimulation was also found to reduced SEB but only compared to the Supplementary motor area stimulation
Barrios et al., 2008	Assign egotistic or moralistic adjectives that are either positive or negative to the self or best friend	TMS to the MPFC significantly reduced SEB but only for egotistic words	No self-enhancement effect was found among their all-female samples
Luber et al., 2012	Assign desirable and undesirable adjectives to either the self or their best friend	real stimulation over the MPFC reduced SEB compared to sham	TMS over the parietal cortex did not affect the self-enhancement effect
De Pisapia et al., 2019	Assign positive and negative adjectives to the self, close other, and the Eiffel Tower or count the number of syllables.	rTMS to the MFPC resulted in inhibition of negative self-evaluation.	(1) TMS reduced the BOLD signal in the MPFC in other condition more than self; (2) TMS increased PCC BOLD signal in negative > positive; (3) TMS over the MPFC increased the BOLD signal in the bilateral IPL only for negative adjective assignment to the self
**tDCS studies**			
Neutral			
Schäfer and Frings, 2019	Recall previously learned word associations with the self, an other, and a neutral object	anodal VMPFC with cathodal DLPFC had no effect in all conditions	N/A
Affective			
De Raedt et al. (2017).	Respond “true” or “false” to positive or negative statements related to the self. Then listened to the negative statements in audio format	anodal tDCS over the DLPFC with cathodal r-SOA reduced negative self-evaluation compared to sham	participants reported being more tired, less vigorous, and less cheerful after both real and sham tDCS
Dedoncker et al. (2019).	Female participants listened to critical, neutral, and positive comments about them. Also reported their perceived level of criticism in their life.	Anodal left DLPFC stimulation reduced emotional responsiveness (measured by functional connectivity) toward criticisms in females with a high level of perceived criticism	Participants reported more fatigue, less vigor, and less cheerful after both real and sham tDCS Participants reported more anger and more depressed after being criticized
Mainz et al. (2020)	Respond descriptiveness of positive and negative adjectives related to the self. Then asked to recall the adjectives regardless of valence	anodal MPFC with cathodal near Oz had no effect for both conditions	Participants exhibited self-enhancement bias toward positive words

*Abbreviations: SPE, Self-processing effect; SEB, self-enhancement bias; SOA, supraorbital area; PCC, posterior cingulate cortex. See ‘Abbreviations’ under Table 1 for missing abbreviations.*

**TABLE 4:** Summary of results of the included NV-SRP studies.

Study	Task	Main Results	Other Results
**TMS studies**			
Self-other discrimination			
Uddin et al., 2006	Presented with pictures of their own face gradually morphed into a familiar other, then press a button to indicate a change of identity	rTMS over the right IPL increased propensity to judge faces to be one’s own	No effect was found in the left IPL stimulation condition
Heinisch et al., 2011	Similar to Uddin et al., 2006 but: (1) used video morphing instead of pictures. (2) Added an unfamiliar face condition. (3) Added a questionnaire on perception of their own body.	rTMS over the right IPL and right DLPFC increased propensity to detect self-faces emerging from famous face but not unfamiliar face.	rTMS over the right DLPFC reduced self-recognition sensitivity in people who have negative attitudes toward their own face
Heinisch et al., 2012	Similar to Heinisch et al., 2011, but measured attention during the task	Replicated Uddin et al., 2006 and Heinisch et al., 2011. But rTMS over the right IPL have no effect on other-other discrimination	Attention had no impact on the effect of right IPL rTMS
Rubber hand illusion (RHI)			
Tsakiris et al., 2008	RHI, PD measurement	Single-pulse TMS over the r-IPL reduced PD when viewing the rubber hand, but increased drifts when viewing the neutral object	N/A
Kammers et al., 2009	RHI, PD measurement, and questionnaire about subjective RHI experience. Immediate and delayed effects were both measured	For immediate effects, rTMS over the left IPL reduced PD when viewing the rubber hand. No difference in subjective experience between real and sham TMS groups	No effect was found for delayed effects of rTMS
Wold et al., 2014	RHI with button clicking to indicate RHI onset, PD, subjective rating of RHI intensity	rTMS over the EBA increased PD during synchronous stroking compared to the asynchronous stroking	No rTMS effect on subjective reports of body ownership
della Gatta et al., 2016	RHI, PD measurement, and questionnaire about subjective ownership.	Single-pulse TMS over the M1 reduced MEP, increased PD, and increased sense of embodiment in the synchronous condition compared to the asynchronous condition	The reduction of MEP increased overtime
Karabanov et al., 2017	RHI procedure where the rubber hand can be anatomically implausible (ownership) and/or detached from real hand (agency). PD, subjective rating of agency and ownership, and the effective connectivity between brain regions were measured.	Single-pulse TMS over the M1 increased PD and ownership. No change of PD and subjective rating induced by paired-pulse stimulation (M1-aIPS)	TMS over the aIPS inhibited motor-evoked potential (MEP) from TMS-induced signals from M1. Such effect is dampened during sensorimotor conflicts
Bassolino et al., 2018	RHI procedure in virtual reality with PD, ownership, and agency measurement	Pulses of supra-threshold TMS over the M1 increased sense of ownership for synchronous stroking compared asynchronous Supra-threshold TMS over the M1 increased ownership and agency compared to sub-threshold for synchronous stroking	No effect was found for perceived agency, disownership, and location when compared the two supra-threshold conditions
Fossataro et al., 2018	RHI procedure in virtual reality with PD and embodiment questionnaire	rTMS over the M1 increased sense of embodiment and disembodiment for synchronous stroking	N/A
Peviani et al., 2018	RHI procedure in virtual reality with PD and ownership questionnaire	rTMS over the VPMC reduced PD without influencing the sense of ownership	N/A
Interoception			
Pollatos et al., 2016	Heartbeat and respiration counting task with interoceptive sensibility questionnaire before and after the task	cTBS over the S1 reduced cardiac IAc compared to occipital stimulation cTBS over the right insula reduced cardiac and respiratory IAc compared to occipital stimulation	Stimulation over the right insula reduced confidence in cardiac IAc compared to occipital stimulation Stimulation over the right insula reduced confidence in respiration IAc compared to S1 stimulation
**tDCS studies**			
Self-other discrimination			
Payne and Tsakiris, 2017	Similar to Heinisch et al., 2011, without the attention task	Anodal stimulation at CP6 with cathode at the vertex decreased propensity to judge faces to be one’s own	N/A
Rubber hand illusion (RHI)			
Convento et al., 2018	RHI, PD measurement, and questionnaire about subjective RHI experience with an additional experiment with no stroking	Anodal stimulation over the right IPL increased PD in synchronous stroking compared to asynchronous stroking, while the effects of anodal right PMC stimulation on PD was indifferent of synchrony	Stimulation to the right IPL not the right PMC induced subjective feeling of “illusory touch” Anodal tDCS to right IPL and right PMC increased PD even without stroking
Hornburger et al., 2019	RHI procedure where location of rubber hand become increasingly anatomically implausible, PD, and subjective questionnaire measurements	Cathodal tDCS facilitated the subjective experience but not PD during RHI compared to the anodal group	Regardless of stimulation, RHI strength and PD exhibited gradual decreases as the rubber hand moved further away from the real hand
Lira et al., 2018	RHI, PD measurement, and questionnaire about subjective RHI experience	anodal tDCS over the PPC but not the PMC facilitated of RHI and subjective reports, regardless of synchrony.	PPC tDCS’s strength of effect in PD is higher in synchronous condition compared to the asynchronous condition
Interoception			
Sagliano et al., 2019	Heartbeat counting task with ECG recordings before and after tDCS	sham tDCS over the left and right insula improved counting accuracy of heartbeats but not real stimulation.	No effect of tDCS on state anxiety

*Abbreviations: PMC, premotor cortex; VPMC, ventral premotor cortex; PD, proprioception drift; ECG, electrocardiogram; IAc, interoceptive accuracy; see ‘Abbreviations’ under Table 1 for missing abbreviations.*

**TABLE 5:** Summary of methodological qualities of the included studies.

Study	Bias arising from the randomization process	Bias due to deviations from intended interventions	Bias due to missing outcome data	Bias in measurement of the outcome	Bias in selection of the reported result	Overall risk-of bias judgment
V-SRP						
Lou et al., 2004 (TMS portion)	L	SC	L	SC	SC	SC
Lou et al., 2010	L	L	SC	L	SC	SC
Schäfer and Frings, 2019	L	L	SC	L	SC	SC
Kwan et al., 2007	L	L	L	L	SC	SC
Barrios et al., 2008	L	L	L	SC	SC	SC
Luber et al., 2012	L	L	SC	L	SC	SC
Mainz et al., 2020	L	L	L	L	SC	SC
De Raedt et al., 2017	L	L	L	L	SC	SC
Dedoncker et al., 2019	L	L	L	SC	SC	SC
De Pisapia et al., 2019	L	L	L	L	SC	SC
NV-SRP						
Uddin et al., 2006	L	L	L	L	SC	SC
Heinisch et al., 2011	L	L	L	L	SC	SC
Heinisch et al., 2012	L	L	L	L	SC	SC
Payne and Tsakiris, 2017	L	L	L	L	SC	SC
Tsakiris et al., 2008	L	SC	L	L	SC	SC
Kammers et al., 2009	L	L	L	L	SC	SC
Wold et al., 2014	L	L	SC	L	SC	SC
Karabanov et al., 2017	L	SC	L	L	SC	SC
Convento et al., 2018	L	L	L	L	SC	SC
Bassolino et al., 2018	L	L	L	L	SC	SC
della Gatta et al., 2016	L	SC	L	L	SC	SC
Fossataro et al., 2018	L	L	L	L	SC	SC
Hornburger et al., 2019	L	L	L	L	SC	SC
Lira et al., 2018	L	L	L	L	SC	SC
Peviani et al., 2018	L	SC	L	L	SC	SC
Sagliano et al., 2019	L	L	L	L	SC	SC
Pollatos et al., 2016	L	L	L	L	SC	SC

**FIGURE 2:**
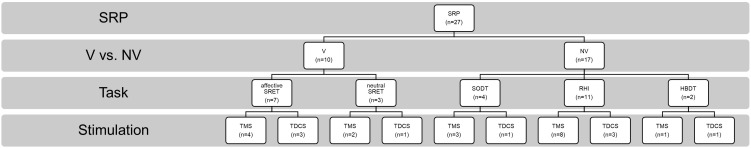
Summary of the included studies by type, task, and stimulation modality. SRP, self-referential processing; V, verbal SRP; NV, non-verbal SRP; SRET, self-referential encoding task; SPE, self-processing effect; SODT, self-other discrimination task; RHI, rubber hand illusion; HBDT, heartbeat detection task.

The included studies are subcategorized into V-SRP or NV-SRP studies based on the broad nature of the task and further categorized based on specific task types. Studies that investigated responses to self-trait adjectives using self-referential encoding task (SRET) were considered within the V-SRP category. SRET studies were further subcategorized into those that used valenced words and therefore assessed the self-enhancement bias (SEB), defined as the tendency toward positive self-evaluation, or self-criticism, defined as the tendency toward negative self-evaluation, and those that selected primarily “neutral” trait adjectives and therefore assessed the self-processing effect (SPE), defined as one’s tendency to process information differentially based on its degree of relevance toward oneself. In comparison, studies that broadly involved tasks involving BSC were categorized into the NV-SRP category (for a definition of BSC, see Park and Blanke, 2019). These NV-SRP tasks were further subcategorized into tasks that investigated one of two forms of exteroceptive NV-SRP or BSC, specifically, (1) visual self-other discrimination task (SODT) or the (2) rubber hand illusion (RHI), or involved (3) interoception in the form of heart-beat detection task (HBDT) or breath counting. In the visual self-other discrimination tasks, participants’ faces were digitally morphed into another face (close others or famous persons) and participants were asked to react to the change of identity during the morphing process. For RHI tasks, studies introduced visual-tactile illusions where participants’ real hand is stroked with a brush in synchrony with a rubber hand to create illusory tactile sensations measured by proprioceptive drift and subjective reports of a sense of ownership of the rubber hand (Botvinick and Cohen, 1998). Finally, HBDT objectively measured heart rate and/or respiration rate and determined participants’ accuracies in self-monitoring these measures over a specified time (Dale and Anderson, 1978; Schandry, 1981; Brener and Kluvitse, 1988; Brener and Ring, 2016). The number of studies identified involving each of these tasks is noted in Figure 2, further categorized as to the form of NIBS that was employed.

## Results

Among the 27 studies, 10 were classified as V-SRP studies and 17 were NV-SRP studies. With regards to stimulation methods, 9 used single-pulse TMS (5 V-SRP, 4 NV-SRP), 9 used rTMS (1 V-SRP, 8 NV-SRP), and 9 used tDCS (4 V-SRP, 5 NV-SRP). The breakdown of the included studies by their method is summarized in Figure 2. The methodological details of each study are summarized in Table 1. Study findings are summarized in Tables 1, 3 for V-SRP and Tables 2, 4 for NV-SRP. Additionally, the studies excluded from this review and the detailed results of each study are described in the appendix (Supplementary Material).

The results of the methodological quality evaluation are listed in Table 5. In brief, the included studies have generally low levels of bias due to randomization, valid interventions, and appropriate use of missing data and outcome measurements. However, all studies received “some concerns” as the overall rating primarily due to the lack of pre-registered plans, albeit some of the papers were published before pre-registrations policies were available (Table 5).

### V-SRP

#### Self-Processing Effect (SPE)

##### TMS studies

**FIGURE 3:**
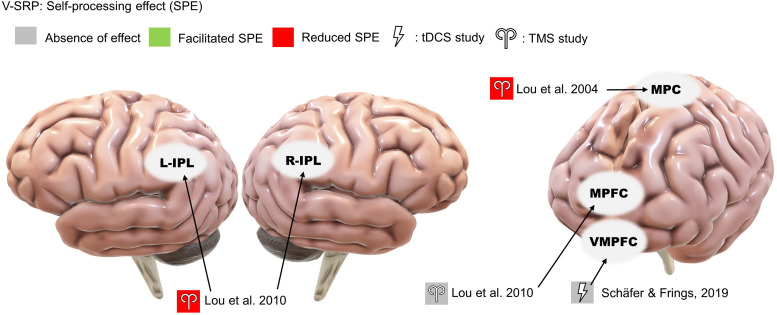
Results of the reviewed studies on neutral SRP with tasks involving self-processing effect (SPE).

Two studies found that single-pulse TMS over the medial parietal region (Pz according to the 10-20 system) and the bilateral IPL reduced SPE (Lou et al., 2004, 2010; Figure 3). In comparison, neither study found involvement of the MPFC during trait-assignment tasks. In addition to behavioral measures, Lou and colleagues (2004) obtained participants’ cerebral blood flow (CBF) with PET scan and showed that TMS application over Pz at 160 ms post-stimulus decreased the CBF in the left IPL more when the words presented were self-related rather than other-related (Lou et al., 2004).

##### tDCS studies

Only a single study investigated the effects of offline tDCS on V-SRP using neutral stimuli, thus examining the SPE (Figure 3). Here, Schäfer and Frings (2019) tested the effects of anodal stimulation over the ventral medial prefrontal cortex (VMPFC) (with cathode over the DLPFC) on participants’ memory of emotionally neutral word associations but failed to identify any effect on V-SRP as the result of this stimulation.

#### Self-Enhancement Bias (SEB) and Self-Criticism

##### TMS studies

**FIGURE 4:**
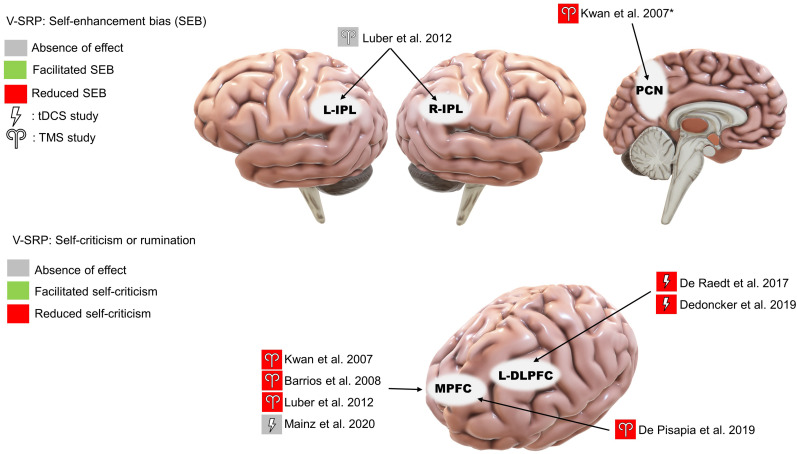
Results of the reviewed studies on emotional SRP with tasks involving self-enhancement bias (SEB) and self-criticism or rumination. *Note: This result was only significant compared to supplementary motor area stimulation.

Four studies consistently found that TMS over the MPFC reduced SEB, supporting the MPFC’s role in emotional SRP (single-pulse: Kwan et al., 2007; Barrios et al., 2008; Luber et al., 2012; rTMS: De Pisapia et al., 2019; Figure 4). Evaluating midline parietal cortex stimulation, Kwan et al. (2007) also found that stimulation applied to the Pz 10-20 EEG electrode site reduced SEB compared to TMS of the supplementary motor area (SMA), although the effect of Pz stimulation was not significantly different from sham stimulation. This complicates interpretation since we cannot conclude that SMA stimulation improved SEB based on the non-significance between SMA stimulation and sham stimulation, albeit this trending result may help future studies in power calculation. Additionally, De Pisapia et al. (2019) reported increased BOLD signal in the PCC in response to MPFC stimulation. However, no significant effect was found for left or right IPL stimulation on SEB (Luber et al., 2012).

##### tDCS studies

Among the three studies that used emotionally valenced stimuli, two studies targeting the left DLPFC reduced negative self-evaluation (De Raedt et al., 2017; Dedoncker et al., 2019; Figure 4) and, in terms of associated mood changes, participants in both studies reported feeling less vigorous and less cheerful after the stimulation. Moreover, Dedoncker et al. (2019) found that the reduction in negative self-evaluation was associated with reduced functional connectivity between the DLPFC and the left posterior insula. In contrast, the only study targeting the MPFC found no effect of offline tDCS on positive or negative self-evaluation (Mainz et al., 2020).

### NV-SRP

#### Self-Other Discrimination

##### TMS studies

**FIGURE 5:**
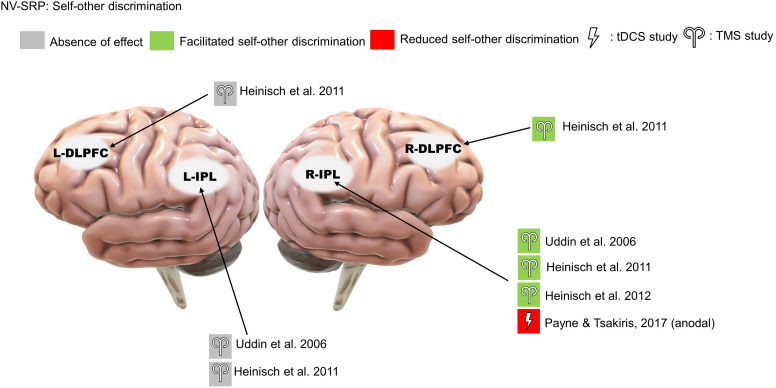
Results of the reviewed studies on NV-SRP with tasks involving self-other discrimination.

Three rTMS studies on self-other visual discrimination consistently found that right IPL stimulation increased participants’ propensity to judge ambiguous faces to be their own (Uddin et al., 2006; Heinisch et al., 2011; Heinisch et al., 2012; Figure 5). Importantly, Heinisch and colleagues (2011, 2012) tested this effect to be self-other specific rather than simply about face-discrimination in general by controlling for face familiarity and other-other discrimination. Further, they found that rTMS over the right DLPFC reduced the judgment bias towards their faces in people who have negative attitudes toward their face, suggesting a role for valenced NV-SRP in the right DLPFC. As for studies that targeted the left IPL, neither Uddin et al. (2006) nor Heinisch et al. (2011) found a significant effect of left IPL stimulation on self-other discrimination.

##### tDCS studies

We identified only a single tDCS study on visual self-other discrimination that found that offline anodal stimulation to the right IPL increased the amount of self-face needed for self-recognition, effectively reducing participants’ bias towards their face (Payne and Tsakiris, 2017; Figure 5).

#### Rubber Hand Illusion

##### TMS studies

**FIGURE 6:**
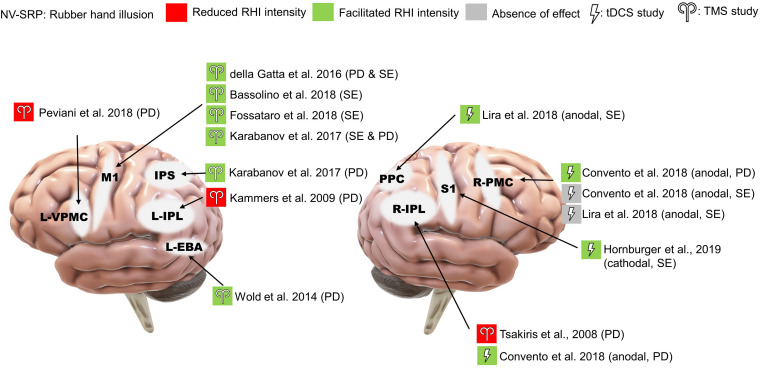
Results of NV-SRP studies on RHI. PD, proprioceptive drift; SE, subjective embodiment of RHI.

The effect of TMS on RHI has been the most studied, with different targets of stimulation. Within these studies, two targeted the IPL and found that TMS reduced RHI-induced proprioceptive drift (single-pulse: Tsakiris et al., 2008; rTMS: Kammers et al., 2009; Figure 6), while one study targeting the extrastriate body area (EBA) found increased proprioceptive drift (rTMS: Wold et al., 2014). Another study using paired-pulse TMS targeting the anterior IPS (aIPS) and primary motor cortex (M1) found numerical but non-significant increases in proprioceptive drift when participants experienced agency and ownership over the rubber hand (Karabanov et al., 2017).

In comparison with the studies that targeted the right IPL, four studies targeted the M1 with TMS and consistently found increases in RHI strength measured by increased proprioceptive drift, sense of ownership and embodiment (single-pulse: della Gatta et al., 2016; Karabanov et al., 2017; Bassolino et al., 2018; rTMS: Fossataro et al., 2018; Figure 6). Interestingly, one study targeting the ventral premotor cortex (VPMC) also found reduced proprioceptive drift without changes in subjective ownership (rTMS: Peviani et al., 2018). These studies suggest that the RHI may be mediated by neural processes on different levels.

##### tDCS studies

Convento et al. (2018) showed that anodal stimulation to both the right IPL and the right PMC increased proprioceptive drift (Figure 6). Interestingly, in their experiment, the effects of tDCS on right PMC were indifferent to synchrony of stroking. Moreover, another study found that online anodal tDCS over the posterior parietal cortex (PPC) but not the PMC facilitated proprioceptive drift and subjective ownership, further supporting the functional segregation between the parietal cortex and the PMC during RHI (Lira et al., 2018; Figure 6). Finally, a study found that online cathodal tDCS over the primary somatosensory cortex (S1) facilitated the subjective experience of RHI when compared to the anodal group but not on proprioceptive drift (Hornburger et al., 2019; Figure 6).

#### Interoception

##### TMS studies

**FIGURE 7:**
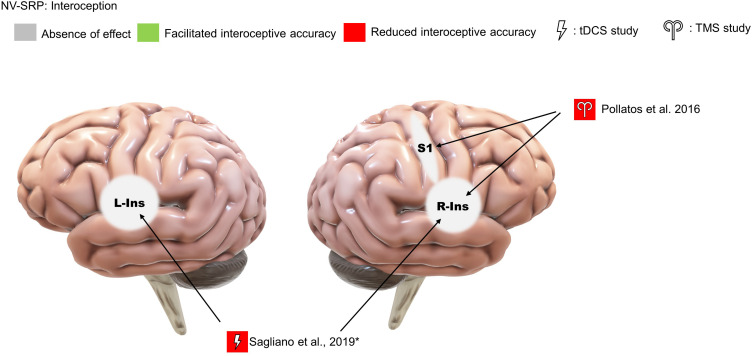
Results of NV-SRP studies on interoception. *Note: This study found that sham but not real stimulation improved interoceptive accuracy.

We identified only one TMS study that investigated the effects of offline continuous theta-burst stimulation (cTBS) on interoception, focusing on right insula and S1 stimulation in comparison to occipital cortex stimulation as a control (Pollatos et al., 2016; Figure 7). The researchers found that right insula and S1 stimulation reduced interoceptive accuracy (IAc), IAc confidence, and interoceptive sensibility. Specifically, cTBS over S1 reduced cardiac IAc while cTBS over the right insula reduced both cardiac and respiratory IAc. Further, in terms of IAc confidence, right insula cTBS reduced confidence in respiration IAc compared specifically to occipital stimulation and reduced cardiac IAc confidence compared specifically to S1 stimulation. Additionally, both insula and S1 stimulation resulted in an increase in self-reported interoceptive sensibility compared to pre-stimulation. Note that one limitation of this study is that the cTBS targeting the insula would have an impact on the overlying frontotemporal cortices, complicating interpretation.

##### tDCS studies

We also identified only a single study that investigated interoception with tDCS. Specifically, Sagliano et al. (2019) found no effect of offline anodal tDCS over the left and right insula on heartbeat counting accuracy (Figure 7). However, sham tDCS facilitated counting accuracy when pre- and post-stimulation performances were compared. The authors suggested that this can be explained by real tDCS reducing the “practice effect” on interoceptive accuracy improvements, concluding that their study supports the role of the insula in IAc.

## Discussion

We systematically reviewed 27 studies that investigated the effect of NIBS on SRP, separated by verbal (V-SRP) vs. non-verbal (NV-SRP) domains. Within the context of V-SRP, studies examined neutral (SPE) vs. emotionally salient (SEB) trait characteristics with SRETs. As described in Tables 1, 3 referring to V-SRP and Tables 2, 4 referring to NV-SRP, the studies described in this review used diverse methods particularly in stimulation type (repetitive: rTMS or event-related: single or pair-pulse TMS) and strength (TMS strength and tDCS current density). In terms of experimental tasks, studies involved either self vs. non-self stimulus discrimination (V-SRP and NV-SRP), response to the rubber hand illusion (NV-SRP), or interoception (NV-SRP). Overall, the methodological quality of the studies reviewed has generally low biases but revealed some concerns. Despite such differences in methods, the results of the reviewed studies revealed some consistencies, albeit with some caveats.

### Verbal SRP (V-SRP)

The results of NIBS on V-SRP were relatively consistent across the 10 reviewed studies in demonstrating a likely role for the cortical midline structures and particularly the left IPL in the self-processing effect (SPE) which, as a task involving self-endorsement responses to relatively neutral adjectives, negates the relevance of emotional valence (Figure 3). Moreover, although Lou et al. (2010) found that TMS to both the left and right IPL resulted in a reduction in SPE, the effect of left IPL stimulation was found to be greater than right IPL, which is in line with fMRI studies on V-SRP such as that of Davey et al. (2016) who found the involvement of the bilateral IPL in V-SRP with the left IPL showing increases in BOLD signal more than the right IPL. However, so far only two TMS studies have investigated the effects of IPL stimulation on V-SRP tasks, and therefore more studies are needed for further validation.

Further, whereas the IPL has been implicated in neutral V-SRP or the SPE, the MPFC demonstrates significance when studies considered emotional valence as a variable (Figure 4). In our review, three single-pulse TMS studies found that MPFC stimulation reduces self-enhancement bias (SEB), although one tDCS study failed to provide corroborative evidence. Additionally, other regions of interest (ROI) such as the precuneus and bilateral IPL received weak support (Kwan et al., 2007; Luber et al., 2012; De Pisapia et al., 2019). The effects of MPFC stimulation on SEB seem to be self-specific and egotistic, referring to an inflated sense of self-worth, status, and power, indicative of an increased SEB (Barrios et al., 2008). Importantly, three studies found that rTMS or anodal tDCS over the MPFC or the DLPFC also reduced negative self-evaluation (self-criticism), suggesting that activation of the prefrontal neurons could have resulted in an overall dampening of emotional response to V-SRP (De Raedt et al., 2017; Dedoncker et al., 2019; De Pisapia et al., 2019). However, Schäfer and Frings (2019) failed to find an effect of anodal VMPFC with cathodal DLPFC tDCS on neutral SRP (i.e., the SPE), while Mainz et al. (2020) failed to find an effect of anodal MPFC with cathodal parietal cortex tDCS on emotional SRP.

Considering the V-SRP studies together, a pattern of functional segregation seems to emerge between the left IPL and the MPFC. Results suggest that the left IPL may be involved in determining the self-relevance of verbal information as primarily tested by the neutral V-SRP studies of SPE (Figure 3), while the MPFC might be more so involved in the affective evaluation of such information as tested primarily by the emotional SRP studies of SEB (Figure 4), consistent with several functional network models of SRP (Fingelkurts et al., 2016, 2020; Frewen et al., 2020). Further, considering the midline posterior cortex, Lou et al. (2004) and Kwan et al. (2007) applied TMS over Pz and found smaller degrees of impact on V-SRP compared to the MPFC, while De Pisapia et al. (2019) found that MPFC had an impact on both the PCC and the bilateral IPL BOLD signals during emotional V-SRP. Interestingly, the dynamic causal modeling conducted by Davey et al. (2016) suggested that the PCC may be the drive for self-related processes with the MPFC as the moderator. Taken together, this supports the notion that although the PCC might be the drive for SRP in general, V-SRP may be more closely related to the MPFC, especially when V-SRP is emotionally significant.

### Non-Verbal SRP (NV-SRP)

Given our affinity to faces even from infancy, being able to distinguish one’s face from others’ faces can be considered as a basic form of NV-SRP, measured by SODTs. In this review, three TMS studies and one tDCS study supported the right IPL’s causal role in self-other face discrimination (Figure 5), confirming the correlational findings from neuroimaging studies (Uddin et al., 2006; Heinisch et al., 2011, 2012; Payne and Tsakiris, 2017). On the contrary, stimulation over the left IPL did not yield any significant change in visual self-recognition. This pattern of lateralization in self-other discrimination in the right hemisphere is consistent with existing evidence (Uddin et al., 2005; Bukowski et al., 2020) but recent evidence also supported the involvement of the left hemisphere (Quesque and Brass, 2019). Furthermore, two studies in our review also found involvement of the right IPL in V-SRP, which suggests that the right IPL may be involved in both V-SRP and NV-SRP (Lou et al., 2010; De Pisapia et al., 2019). Interestingly, Heinisch et al. (2011) found that stimulation over the right DLPFC also reduced visual self-recognition but only in people who have pre-existing negative attitudes toward their own face, effectively reducing their negative self-evaluation. Therefore, there might be some degree of laterality in NV-SRP in the right hemisphere, although contrary evidence also exists. It is possible that NV-SRP is associated with multiple processes and therefore affected by stimulation to the right IPL primarily, and other regions such as the left IPL and the DLPFC to some degree. Considering the right IPL as part of the MTL subsystem of the DMN, one might postulate that NV-SRP partially overlaps with the functions of the MTL subsystem and interacts with affective processes in the PFC, which may explain the results of Heinisch et al. (2011). Given that most studies on emotional SRP have focused on V-SRP instead of NV-SRP, future studies could also investigate the effect of NIBS on emotional NV-SRP with MPFC stimulation, for example, in response to facial displays of emotion or using a priming methodology (Frewen et al., 2013, 2017, 2020).

Contrary to the possible right hemisphere dominance in visual self-other discrimination, NIBS over both the left and right hemispheres altered the effects of RHI for the contralateral hand (Figure 6). It is important to note that RHI strength has two dimensions: the change in perceived hand position measured by proprioceptive drift, and the change in subjective experiences such as embodiment and ownership of the rubber hand. As illustrated in Figure 6, stimulation over different areas had a differential impact on proprioceptive drift and subjective experience. We found that TMS over the M1 and the EBA facilitated subjective experience, whereas TMS over the left IPL and the left PMC reduced proprioceptive drift. Additionally, anodal tDCS over the right PMC and the right IPL facilitated proprioceptive drift, and cathodal tDCS over the S1 facilitated subjective experience (Figure 6). These results may offer support for hierarchical processing in the RHI wherein low-level somatosensory processing might be relayed to high-level multisensory integration to form feelings of ownership and agency over the body (Apps and Tsakiris, 2014). Consistent with this interpretation, paired-pulse TMS over the aIPS reduced the motor-evoked potentials from M1 (Karabanov et al., 2017) that was dampened by sensorimotor conflict, supporting the “comparator” mechanism that processes incoming sensory and proprioceptive inputs as proposed by Tsakiris (2010). In our review, areas shown to affect proprioceptive drift include the left VPMC, IPL, EBA, M1, and right IPL for proprioceptive drift, while areas shown to affect subjective experience included left M1, right PMC, S1, and the PPC. According to the hierarchical theory, the right IPL and the PPC might act as the integration area for proprioceptive drift and subjective experience respectively, but such assumptions need to be validated by further evidence.

As compared to the RHI, which involves the processing of one of the bodily extremities, interoception can be measured from a sensory perspective toward internal bodily sensations by IAc of heartbeat or respiration and a subjective perspective by interoceptive sensibility and IAc accuracy. With regards to accuracy, both of the reviewed NIBS studies supported the causal role of the left and right insula in both cardiac and respiratory interoception (Figure 7; for the right insula: Pollatos et al., 2016; and for the left and right insula: Sagliano et al., 2019). Further, with regards to subjective experience, Pollatos et al. (2016) found the involvement of the right S1 in both IAc and the awareness associated with IAc, suggesting that S1 may also be part of a neural system that links interoceptive sensory signals with awareness of such signals. These results provided support for the existence of Park and Blanke’s (2019) integrative BSC system connecting multiple interoceptive sensory areas. Referring to meta-awareness as measured by IAc confidence, Pollatos et al. (2016) also argued that the decline might be related to disturbance of the sensory integrative processes in the anterior insula, resulting in mismatching between beliefs and sensory input. However, a more comprehensive picture of the brain areas involved still requires further evidence, as the NIBS literature on IAc and awareness is scarce.

Overall, our review provides causal support for brain regions discovered by neuroimaging studies in NV-SRP in the parietal cortex (including the IPL and PPC), the insula, and sensorimotor cortical areas (including the M1, S1, PMC, and EBA). More importantly, both interoception and BSC (observed in RHI studies) were able to show that stimulation to NV-SRP-related areas can induce changes in participants’ perception of internal or external stimuli such as proprioceptive drift or IAc, and they can also alter participants’ subjective experiences measured by self-reports, supporting the existence of a common NV-SRP system proposed by Park and Blanke (2019). In their theory, self-identification is associated with a PMC-IPS-insula network whereas self-location is associated with a PCC-IPS network. While a number of NIBS studies investigating the response to the RHI were able to alter self-location by stimulating the IPL, no reviewed NIBS studies on self-identification have chosen the PMC or the insula as the stimulation target, which can be of interest for future studies. Moreover, most of our reviewed NV-SRP NIBS studies have targeted the sensorimotor areas, which may be lower within the hierarchy of processes producing the subjective experiences associated with NV-SRP. In the study conducted by Karabanov et al. (2017), paired-pulse TMS was used to investigate the modulatory role of a higher-order integrative area (e.g., aIPS) toward the M1; future NIBS studies may use similar experimental paradigms to investigate the modulatory relationships between ROIs in NV-SRP.

However, as compared to the response to visual self-recognition tasks, we did not observe a strong right hemisphere dominance for RHI studies wherein left IPL, left EBA, and left VPMC stimulation all showed significant effects on proprioceptive drift (Figure 6). One explanation is that compared to self-identification and IAc which do not involve processing only of one side of the body, RHI tasks are more complex, involving multiple processes from raw sensorimotor processing and proprioception of one-sided bodily stimuli (e.g., left or right hand) to a higher-level integration into subjective experiences and BSC as a whole. However, these conclusions should be treated with caution since only two NIBS studies were found in the interoception category, one of which showed bilateral response in the insula (Figure 7; Sagliano et al., 2019). Therefore, future studies may investigate the effect of NIBS over higher-order parietal regions on NV-SRP and compare unilateral to bilateral montages.

### Limitations and Future Directions

A quantitative meta-analysis was not possible for this review due to the large variability of study designs; thus, we relied on a qualitative and descriptive approach. Another limitation is that the quality of methodology utilized was judged to have some concerns for several of the included studies in this review; future studies are encouraged to utilize stronger methodology, ideally pre-registering their study and including double-blinded designs including both sham and active stimulation controls. Moreover, sample sizes in many studies were small and underpowered, and participant samples were frequently not well described such as for demographic characteristics, a problem that also requires attention in future studies.

In addition to the small number of NIBS studies that have investigated SRP, most reviewed studies have only investigated the effect of NIBS on subjective and behavioral outcomes. From a practical perspective, self-report and behavioral measures can have direct clinical applications, although the underlying brain mechanisms of NIBS on SRP remain a “black box” until the effects of NIBS are routinely simultaneously investigated not only for phenomenological and behavioral outcomes but also for neurobiological outcomes (e.g., EEG, fMRI). Moreover, the experimental tasks used in NIBS studies exhibit a clear verbal vs. non-verbal split between studies, while no studies have so far compared the response to both V-SRP and NV-SRP in the same study. Therefore, future studies may comparatively investigate both verbal and non-verbal aspects of SRP under one experimental design, and compare the effects of different stimulation sites, for example, inter-hemispherically within the IPL or the insula, or between posterior (e.g., IPL, PCC) and anterior (e.g., MPFC) sites, as well as by stimulation method (e.g., TMS vs. tDCS). Moreover, in so far as it is well known that many psychiatric and neurological disorders are associated with disturbances in SRP (e.g., reviewed by Frewen et al., 2020), it will be important to evaluate whether NIBS during SRP tasks would have any clinical significance in treatment, for example, for reducing self-criticism associated with affective disorders such as depression and posttraumatic stress.

## Author Contributions

ZB, BH, and PF contributed to the conception and design of the study. BH drafted the framework and the initial version of the article. ZB and PF revised and expanded on the article. AB edited and offered suggestions on the article. All authors contributed to the article and approved the submitted version.

## Conflict of Interest

The authors declare that the research was conducted in the absence of any commercial or financial relationships that could be construed as a potential conflict of interest.
