# Supplementary material for: Self-Referential Processing Effects of Non-invasive Brain Stimulation: A Systematic Review
Source: Front Neurosci. 2021 Jun 10;15:671020. doi: 10.3389/fnins.2021.671020 (PMC8223877; doi:10.3389/fnins.2021.671020)
Supplement: Supplementary file 1 [file Data_Sheet_1.DOCX]

Supplementary Material

# Supplementary Tables

| Supplemental Table 1 |
| --- |
| *List of excluded studies* |
| Study excluded because task did not involve SRP |
| Bellis, Trojano, Errico, Grossi & Conson, 2017 |
| David et al., 2009 |
| Guise et al., 2007 |
| Hu et al., 2017 |
| Liepelt et al., 2016 |
| Martin, Dzafic, Ramdave & Meinzer, 2017 |
| Mondino, Poulet, Suaud-Chagny & Brunelin, 2016 |
| Pisoni, Lauro, Vergallito, Maddaluno & Bolognini, 2018 |
| Preston & Newport, 2008 |
| Schuwerk et al., 2014 |
| Zhang, Chen, Hu & Mai, 2019 |
| Giardina, Caltagirone, Cipolotti & Oliveri, 2012 |
| Salerno et al., 2012 |
| Civai, Miniussi & Rumiati, 2015 |
| Study excluded because only included spontaneous SRP condition (e.g., resting state) |
| Axelrod, Rees, Lavidor & Bar, 2015 |
| Kajimura, Kochiyama, Nakai, Abe & Nomura, 2016 |
| Alon, Roys, Gullapalli & Greenspan, 2011 |
| Baeken et al., 2017 |
| Kajimura & Nomura, 2015 |
| van der Werf, Sanz-Arigita, Menning & van den Heuvel, 2010 |
| Salehinejad, Nejati & Nitsche, 2020  Coulborn, Bowman, Miall & Fernández-Espejo, 2020 |
| McCallion, Robinson, Clark & Witkiewitz, 2020 |

# Summary of each included study

**V-SRP**

***Neutral V-SRP: Self-processing effect (SPE)***

**Lou et al. (2004).**Based on the framework proposed by Gardiner (2001) and neuroimaging evidence (Cabeza & Nyberg, 2000), Lou and colleagues (2004) suggested that the frontal activity in episodic memory retrieval and parietal activity in semantic memory retrieval might be affected by the degree of self-reference. To test this hypothesis, twenty-five participants (7F/6M) completed the self-referential encoding task (SRET) while undergoing single-pulse TMS. In this experiment, participants first rated how applicable personality trait adjectives are to themselves, their best friend, or a famous figure (in this case, the Danish Queen), followed by a retrieval task. During the retrieval task, participants were asked to indicate if a trait had been characterized as applicable to the person as quickly as they can but prioritizing accuracy, and an efficiency score was calculated as the outcome variable to describe both the RT and accuracy performance in this task and counteract the speed/accuracy trade-off. Participants received TMS only during the retrieval task with randomly varying discrete latencies (0, 80, 160, 240or 480 ms) after the onset of word stimulus over one of three locations: 1) Oz for occipital pole; 2) Pz for medial parietal cortex; 3) Fz for frontal cortex at 150% of motor evoked potential (MEP) in the feet.

Overall, task performance supported the self-processing effect (SPE) where efficiency in recalling self-related adjectives was higher than recalling adjectives related to one's best friend, which was then higher than recalling traits about the Danish Queen. Results indicated that TMS significantly inhibited efficiency at 160 ms post-stimulus for the self condition compared with the other condition, and only when applied at Pz. This suggests that the medial parietal/posterior cingulate cortex is at least involved in the SPE. Although there was no significant direct effect of Fz stimulation, the authors suggested that given the strong anatomical connection between the medial frontal and medial parietal regions, the activities and synchrony of these regions might also underlie self-awareness regulation.

**Lou et al. (2010).**Lou et al. (2010) tested the effect of MPFC and bilateral lateral parietal cortex (P3, P4) stimulation on SRP by using a single-pulse TMS applied at various discrete time points (0, 80, 160, 240, or 480 ms) after the stimuli onset at 150% resting motor threshold (RMT) of the left hemisphere. Sixteen (7F/9M) healthy participants were included in a within-subject design where all participants received all three types of TMS. For the SRET, participants rated the degree of descriptiveness of adjectives on themselves or their best friend on a scale of 1 to 6 (*extremely uncharacteristic* to *extremely characteristic*). Then, participants were asked to indicate their choice of reference target (self or other) in the previous task as quickly as possible and the outcome variable was the same efficiency score metric used in Lou et al. (2004). Given the established SPE in MPFC, efficiency in the self condition was predicted to be higher than the best friend condition for MPFC stimulation. Therefore, rather than using a sham control condition, stimulation of the MPFC (Fz) was considered a control condition in this experiment.

Results first found that SPE was not modulated by MPFC stimulation. Specifically, efficiency in the self condition was significantly higher than efficiency in the best friend condition, regardless of the timing of TMS onset. Secondly, stimulation of the left parietal cortex was found to suppress the SPE only when conducted 160ms, 240ms, and 480 ms post-stimulus. This indicated that the SPE was only present when TMS was applied 0 or 80 ms after stimulus onset. Surprisingly, when TMS was applied 160 ms after the stimulus onset, efficiency in assigning traits for the best friend was significantly higher than assigning to the self, which is the opposite of the SPE. For the right parietal cortex, TMS applied at all time points except for 480 ms suppressed SPE, regardless of the referential target. For the first two time points (0 and 80 ms), efficiency in the self condition was significantly higher than efficiency in the best friend condition. In other words, decreased self-reference effects were found at 160, 240, and 480ms.

Based on these results, the researchers concluded that the parietal cortex is important in retrieving self-specific processed information and that the default mode network has an essential role in SRP. The authors' previous experiments have found no effect for a single TMS pulse of the MPFC on the self-reference effect (Lou et al., 2004). Possibly, a single pulse of TMS on the MPFC is not sufficient to alter SRP. The authors also mention that TMS stimulation at a later interval (i.e. > 480 ms) might affect the MPFC. Note that this study did not have a sham-control condition. Their reasoning for this was that a pilot study with no TMS vs. MPFC TMS treatments on a different group of participants found no difference in the SPE between the two treatments. Hence, performance during MPFC TMS was used as a reference. It is also important to note that TMS occurred during the second task after participants judged the adjectives to relate to the self vs. their best friend. Although studying the effects of TMS on the SPE is still relevant, this method focuses on retrieving self-associated information rather than SRP as encoding. Studying the effects of stimulation during the first judgement task would be useful in explaining the role of CMS during the processing of the descriptive adjectives as representing the self or the participant's best friend.

**Schäfer and Frings (2019)** looked at the effects of neuromodulation through tDCS on the VMPFC. They predicted that tDCS of the VMPFC would alter SPE, based on previous findings that suggested a role for SRP in the VMPFC and the processing of others in the DMPFC (Wagner et al., 2012; Denny et al., 2012). A total of 65 participants were included in the study. For the SRET, participants initially learned series of word associations. For example, one participant may be given "*I am the circle, a stranger is the square, and the chair is the triangle*." In this case, they learned the association between “I”, “a stranger”, “the chair” and “circle”, “square”, “triangle”, which were paired randomly. Later during the matching task, participants were presented with scrambled associations (144 trials total) and asked to indicate the correctness of each match. Participants completed this matching task before and after receiving tDCS. Reaction time and accuracy were collected for each item. Once this was completed, participants were either given anodal VMPFC tDCS with cathodal dorsolateral prefrontal cortex (DLPFC), or cathodal VMPFC tDCS (with anodal DLPFC). A current of 0.5 mA was applied for 20 minutes to the VMPFC. Given the size of the electrodes, this results in a current density of 0.056 mA/cm for the active electrodes and a current density of 0.014 mA/cm for the reference electrode. After the tDCS session, participants completed a post-tDCS association task, which followed the same method as the pre-tDCS task. Surprisingly, a self-processing effect (defined as better performance in self-associated matching trials than other-associated trials) was not found after both types of tDCS stimulation (anodal VMPFC & cathodal VMPFC). The results were not in line with previous neuroimaging research, which indicated a correlation between SRP and VMPFC activity. One possible interpretation of this study's findings is that the relationship between the VMPFC is correlational and not causal, suggesting that SRP may rely on the integrated processing of multiple regions in the brain. Another possible explanation for the null result is that a single session of tDCS may not be sufficient to result in any changes in SRP. This could be overcome by measuring the activity in the VMPFC using neuroimaging after the tDCS sessions, allowing researchers to compare if the tDCS resulted in a level of activation comparable to neuroimaging results. It is also worth mentioning that there was a difference in current density between the active and the reference electrode, there might have been an effect of slight facilitation (during cathodal VMPFC) or slight inhibition (during anodal VMPFC) that could have affected the results, as the DLPFC has previously been found to be involved in SRP (Baeken et al., 2017). Nevertheless, the findings of this study require further research before we can confidently rule out a direct role of the VMPFC in self-other discrimination.

***Emotional V-SRP: Self-enhancement bias (SEB) and self-criticism.***

The affective valence is a salient feature of V-SRP that is associated with many neurological and behavioural outcomes (LeMoult, Kircanski, Prasad & Gotlib, 2017; Frewen, Thornley, Rabellino & Lanius, 2017). For positive V-SRP, neuromodulation studies have focused on self-enhancement bias (SEB), which posits that people evaluate themselves more positively and more socially desirable (Krueger, 1998). Studies have also investigated the effects of NIBS on negative V-SRP in the context of self-criticisms. Neuroimaging evidence suggests that MPFC and other CMS structures are correlated with affective V-SRP (Araujo, Kaplan & Damasio, 2013). Below we review the NIBS studies that aimed to investigate the potential causal relationships.

**Kwan et al. (2007).**Given the converging evidence of MPFC's role in self-enhancement bias, Kwan and colleagues (2007) investigated the role of MPFC in SEB with virtual lesions created by single-pulse TMS. Twelve university students (10F/2M) completed the SRET where they were asked to assign positive, neutral and negative adjectives from Anderson's list (1968) to either the self or their best friend. Participants were randomly assigned to receive TMS in one of the four conditions: 1) MPFC stimulation 2) precuneus stimulation at Pz, given its involvement in self-related processing but no evidence for involvement in SEB 3) supplementary motor area (SMA) stimulation, given it has not been found in self-related bodily processes 4) sham stimulation at 90̊ above Cz. Results indicated that only real stimulation over the MPFC reduced SEB compared to sham, supporting MPFC's role in SEB. In addition, precuneus stimulation was also found to reduced SEB but only compared to the SMA stimulation. The authors suggested a potential supporting role of the precuneus in SEB. Given the connection between the MPFC and orbitofrontal regions, the authors also suggested that the MPFC-orbitofrontal circuit is involved in SEB. Since the MPFC-orbitofrontal circuit was found to be involved in emotional processing (Schoenbaum 2004), Kwan and colleagues (2007) suggested that change in affect might underlie SEB, or that SEB can be a form of self-deception given the link between the MPFC and deception of others (Lee et al., 2002).

**Barrios et al. (2008).**This study aimed to replicate the finding of Kwan et al. (2007) with a different form of SRET to studying two different forms of SEB originally proposed by Paulhus & John, (1998): 1) egotistic SEB, characterized by an inflated sense of intelligence, power and status 2) moralistic SEB, characterized by an inflated sense of good character to satisfy the need for acceptance. Barrios and colleagues (2008) chose 32 positive egotistic, 32 negative egotistic, 32 positive moralistic, and 32 negative moralistic words from Anderson's list (1968) for their study and ask ten female participants to assign them to either the self or a close other (their best friend). The sites of TMS in this study are the same as Kwan et al. (2007) (MPFC, Pz, and sham). Reaction time (RT) was also recorded as an outcome measure. Results on SEB indicated TMS to the MPFC significantly reduced SEB but only for egotistic words. The authors suggested that the MPFC might be associated with ego-based self-enhancement. Interestingly, even though Barrios and colleagues (2008) supported the finding of Kwan et al. (2007) that the MPFC plays a role in SEB, they did not find self-enhancement among their samples. The authors proposed that it might be due to their all-female sample, and the impact of gender in self-enhancing tendencies should be investigated by future studies.

**Luber et al. (2012).** The results of Kwan et al. (2007) and Barrios et al. (2008) suggested that the MPFC plays a role in SEB. However, Lou et al. (2004) and Lou et al. (2010) failed to find MPFC's involvement in SPE. One explanation of such findings was that SEB and SPE are processed differently in the brain, perhaps separated by the involvement of emotional valence. As a follow-up, Luber et al. (2012) investigated the effect of single-pulse TMS over the MPFC and the left and right IPL during the SRET where participants were asked to process self-related desirable vs. undesirable adjectives. Similar to Lou et al. (2010), single-pulse TMS was delivered at 0, 80, 160, 240, and 480 ms post-stimulus onset. The researchers used the data of 18 participants in the Lou et al. (2010) study and recruited 27 new participants, which results in a total sample size of 45 (21F/24M). The 27 new participants completed the same tasks with the same adjectives as used in Lou et al. (2010) but received no TMS stimulation. This allowed for a better control group than assuming that MPFC TMS has a null effect (as in the Lou et al., 2010 study). The researchers also differentiated between the self-reference effect of desirable and undesirable words to check if a self-enhancement bias was present. Accordingly, the authors segregated the data of the 18 participants from Lou et al. (2010) between desirable and undesirable adjectives. Participants in this study were asked to indicate "yes" or "no" to present adjectives.

Results indicated that only TMS to the MPFC suppressed the self-enhancement effect in a time-specific manner. The self-enhancement effect showed the greatest suppression when participants assigned more positive traits to their best friends than themselves. The suppression of the self-enhancement effect began to diminish at shorter and longer intervals than 160 ms. Concerning the parietal cortex, no self-enhancement effect was found. Based on the findings of this study and of Lou et al., (2010), the authors concluded that the anterior and posterior portions of the default mode network are responsible for different parts of SRP. The posterior parietal DMN regions might be responsible for the retrieval of associations related to the self, while the anterior portion (i.e. MPFC) more responsible for self-evaluative processing. This explains why no effect was found for TMS of the MPFC when only the retrieval of self-related associations was tested. Frewen and colleagues (2020) suggested that the affective component of SRP can have a positive or negative valence. Likely, the suppression of the self-enhancement effect found during TMS of the MPFC indicates that the MPFC's role in differentiating between positive and negative affect towards the self. Future experiments should investigate if the self-enhancement effect is specialized in sub-regions of the MPFC and if there are any differences during the encoding of positive vs. negative words.

**Mainz and colleagues (2020)** tested the effect of MPFC tDCS on SEB with seventy-five healthy male participants. Participants were randomly assigned to receive 1) anodal Fpz with cathodal Oz or 2) cathodal Oz with anodal Fpz or 3) sham stimulation. After 20 minutes of tDCS, participants completed the SRET and a memory task. During SRET, participants were presented with adjectives that were either positive or negative in valence. Their task was to decide the self-descriptiveness of those adjectives. Later in a memory task, participants were asked to recall as many adjectives from the SRET as possible, regardless of their choice of self-descriptiveness. Participants' choice of words and reaction times were recorded as the outcome measures.

Results confirmed the typical SEB effect where people assign more positive than negative traits to the self. However, anodal tDCS over the MPFC with cathode over Oz did not significantly modulate participants' choice of assignment, nor did it affect their reaction times. The researchers suspected several explanations for the null results. First, it can be the result of the lower-level intensity of tDCS compared to TMS. Second, evidence suggests that gender might affect tDCS efficacy (Lee et al., 2018). Therefore, Mainz and colleagues (2020) suggested that the role of gender should be considered in future studies on SEB. Third, the authors suggested that the effect of tDCS on cognitive performance overall including SRP in healthy subjects remains controversial due to possible ceiling effects (Hoy et al., 2013). Fourth, since most studies with positive results used a self-other discrimination paradigm such as Luber et al. (2012), the reduction of self-referential bias might be specific to the self vs. other contrast rather than self vs. non-self as implemented in this study. Finally, since this study only considered effect sizes of generalized eta-squared over 0.50 to be relevant, increase the strength of stimulation by using TMS or increasing the sample size might allow detection of the relatively weak effect of NIBS on SEB.

**De Raedt et al. (2017).** De Raedt and colleagues (2017) studied changes in self-esteem in response to left stimulation during ruminative self-focus with tDCS. This study assessed ruminative thinking, mood, implicit and explicit self-esteem in 32 healthy right-handed females. During the SRET, participants were asked to respond "true" or "false" to positive or negative statements related to the self. The reaction times to these statements were considered implicit self-esteem, and the given responses are considered explicit self-esteem. Structural MRI scans were obtained for each participant to locate their left DLPFC, which was the site for the anodal electrode. The cathodal electrode was placed over the right supraorbital area. After the structural scan, 1.5 mA real or sham tDCS was applied for 20 mins. After stimulation, participants underwent a criticism paradigm where they heard an equal number of critical, neutral, and praising comments made by a female voice. This is then followed by a resting-state fMRI scan. After rs-fMRI, the same critical audio scripts from the criticism paradigm were then reused and played to participants again before SRET, which was done at the end of the experiment. Ruminative thinking was tested at three time points: before and after tDCS, and after the criticism paradigm.

With regards to mood, participants reported being more tired, less vigorous, and less cheerful after tDCS, but there was no difference in mood change between real and sham tDCS. Participants also reported more anger and less cheerful (approaching significance) after the criticism paradigm. With regard to brain stimulation, researchers found that anodal tDCS over the DLPFC reduced ruminative thinking (MRSI scores) compared to sham. This effect of tDCS still held even after the criticism paradigm. This finding is in line with DLPFC's role in cognitive control over negative emotion. Although the facilitation of DLPFC did not directly influence self-esteem (implicit and explicit), further mediation analysis indicated that this tDCS-induced reduction in ruminative thinking significantly mediates the relationship between DLPFC stimulation and implicit self-esteem, but only after receiving critical comments. Explicit self-esteem was unaffected in either condition. The authors explained that since tDCS only modulates momentary self-esteem, the lack of effect on explicit self-esteem can be explained by the fact that implicit self-esteem is more state-oriented and susceptible to the impact of our emotional state in the present. On the contrary, explicit self-esteem reflects the ideal self, which is in the future instead of the present. Finally, this study is limited by the lack of control condition for criticism (i.e., a "no criticism" condition) and its small all-female sample.

**Dedoncker et al. (2019).** Dedoncker and colleagues (2019) investigated the effect of tDCS and self-referential criticisms on mood and DLPFC resting-state functional connectivity using a similar paradigm as De Raedt et al. (2017). For SRET, forty-one healthy right-handed females listened to critical, neutral, or praising auditory comments directed at them after tDCS. They were instructed to listen carefully and imagine they were overhearing those comments directed at them. For tDCS, participants received 1.5-mA randomized real or sham tDCS for 20 mins in two separate sessions with anode placed at their left DLPFC (identified by structural MRI) and cathode placed at the contralateral supraorbital region. In addition, participants self-reported their overall perceived criticism on a 10-point scale with 1-5 being “low” and 6-10 being “high” (e.g. "How critical do you think people in your nearest environment – family, friends… are of you?", p.1063). Participants' resting-state functional connectivity data was collected three times in each session, before and after both tDCS and the criticism paradigm.

With regards to mood, participants reported more fatigue, less vigour, and less cheerfulness after tDCS regardless of real or sham stimulation. Participants reported more anger and more depressed after being criticized. No difference in mood change was found between people with high vs. low perceived criticism. Resting-state results indicated that for participants with high perceived criticisms, real tDCS to the left DLPFC reduced the functional connectivity between the left DLPFC and the left posterior insula compared to sham. Secondly, critical comments after real tDCS reduced functional connectivity between the left dACC and right DMPFC in participants with high perceived criticisms. Previous studies have shown decreased BOLD activity in response to criticism in areas of the brain associated with monitoring (dACC), evaluating (DMPFC), and regulate (DLPFC) emotional stimuli in depressed patients (Hooley et al., 2009). Taken together, the resting-state data suggested that tDCS over the DLPFC might reduce emotional responsiveness towards criticisms in females with a high level of perceived criticism. However, this study failed to show such a reduction on a behavioural level. Given the dichotomous nature of their measurement of perceived criticism (high vs. low), the authors suggested that future studies investigate the effects of individual trait variability on the brain's response to criticisms.

**De Pisapia et al. (2018).**De Pisapia and colleagues (2018) studied the effect of TMS of the MPFC on affective SRP using functional MRI. Fourteen participants (7M/7F) underwent two sessions of 1-Hz rTMS (real or sham) on the MPFC at an intensity of 100% resting motor threshold. The order of the sessions was randomized for each participant. After 14 minutes of stimulation, participants underwent fMRI where they completed the SRET where they were presented with adjectives and asked to either 1) assign it to the self or 2) assign it to a close other or 3) assign it to the Effiel Tower or 4) count the number of syllables. Participants were told to judge as quickly as they can if the association between the adjective and the referent was relevant, irrelevant, or neutral. The adjectives given were either positively or negatively valenced. Reaction time and blood oxygen level-dependant (BOLD) signals were measured while participants completed the task.

The researchers found that real TMS to the MPFC led to an increase in reaction time when processing negatively valenced self and other associations compared to sham TMS. In other words, TMS deactivation of the MFPC resulted in inhibition of negative self-evaluation. Analysis of BOLD activity in three ROIs revealed that 1) real but not sham TMS reduced the BOLD signal in the MPFC in the other condition compared to the self condition. 2) Real but not sham TMS over the MPFC also increased the BOLD activity in the PCC for assignment of negative adjectives across conditions. 3) Real but not sham TMS over the MPFC increased the BOLD signal in the bilateral angular gyrus only for negative adjective assignment to the self. Based on these results, the researchers concluded that the MPFC plays a rather important role in processing negative attributes of the self and others. The researchers also suggest that the affect and referent dimensions are not independent of each other but are interconnected in the MPFC and in other brain regions that are part of a nodal network. The authors suggested that the findings can be interpreted through motivational attention, where participants perceive the negative stimuli as threatening, leading to a heightened attentional response in the MPFC. The authors suggested that future studies should investigate the role of the MPFC in motivational attention to better understand how this might relate to findings of MPFC activity during SRP. One of the limitations of this study is that participants underwent two rTMS sessions on the same day. Although the two sessions were separated by at least 21 min, carry-over effects cannot be completely ruled out.

**NV-SRP studies**

***Self-other face discrimination***

Self vs. other face discrimination is considered to be an exteroceptive non-verbal form of SRP thought to be at least partially mediated by the right TPJ/IPL (Uddin et al., 2006). Below, we review the NIBS studies that investigated the role of right TPJ in self-other face discrimination.

**Uddin et al. (2006).**Uddin and colleagues (2006) were the first to test the effects of rTMS on self-other face discrimination with a face-morphing task (the self-other discrimination task, SODT). In this task, participants' photographed faces were morphed into their choice of a familiar other (a friend or a colleague). Then, participants viewed faces that were 0%, 20%, 40%, 60%, 80%, and 100% morphed into their friend or colleague's face in random order; in other words, 0% morphed faces constituted faces that were fully their own, 100% morphed faces were those that were fully of the familiar other, and the other degrees listed indicated the photograph partially showed themselves while partially also showing the other person. Participants pressed a button when they detected a change of identity. After the task, participants received 20 min 1-Hz rTMS at either the right or the left IPL followed by the task again so their pre- and post-stimulation task performance and be contrasted. Here, the left IPL treated as a control site because only the right but not left IPL response was consistently identified in neuroimaging studies of own-body perception and self-face recognition (Blanke, Ortigue, Landis & Seeck, 2002).

Results indicated that deactivation over the right IPL but not left IPL resulted in a significant bias toward recognizing one's own face, that is, participants were less conservative or faster in judging a face to be the self after right than left IPL stimulation. Therefore, the authors proposed that self-recognition is specialized in the IPL of the right hemisphere. Moreover, based on prior SRP fMRI studies (Uddin et al., 2005), this finding supports the notion that mirror neurons within the frontal-parietal network in the right hemisphere might underlie self-other recognition and therefore plays an important role in higher social functions.

**Heinisch and colleagues (2011)** also tested the effects of rTMS on self-other face discrimination with an improved experimental paradigm based on Uddin and colleagues (2006) that was subsequently used by several studies (Payne & Tsakiris, 2016; Heinisch, Krüger and Brüne, 2012). In brief, the prior experimental SODT had the limit that photographs always morphed into only one other stimulus, a confound that was resolved in future experiments by morphing participants' photographed faces into either a famous person (recognized by all participants) or several unfamiliar individuals, and trials that morphed either unfamiliar persons or the famous person into the self were also included. Moreover, rather than using discrete images, the morphing steps were made into a video with the progression of each frame representing the morphing process in both directions (self to other and other to self).

Again, before and after rTMS, participants were shown the video depicting the morphing process and they were asked to press a button when they detected a change of identity. Given established roles for the TPJ and the PFC's in face recognition, TMS was applied to the bilateral DLPFC (triangular centers of F3, F7, Fp1 and F4, F8, Fp2) and bilateral TPJ (CP5 and CP6, midpoints of C5, P5 and C6, P6). Each participant received stimulations to those four sites plus a sham stimulation in random order. Participants also completed a self-appraisal questionnaire (FKB-20, translated as “Body Scheme Questionnaire”) related to how they evaluate their own bodies.

Results indicated that self-identification was faster after rTMS to the right TPJ but not left TPJ, which is consistent with the study conducted by Uddin and colleagues (2006). This result supported previous findings indicating right TPJ involvement in self-other discrimination but not left TPJ. However, decreases in RT were found when a video transformed from a famous person to the self, but not from self to the famous person. The authors suggested that this pattern of self-other discrimination follows the typical developmental trajectory of humans and other primates' default assumption: if we see a face, the face is likely from the other instead of the self. We learn to distinguish the self from the other only through repeated exposure to self-images. Another interesting finding is that rTMS over the right DLPFC also reduced self-recognition RT modulated by the valence of evaluation of one's image. That is, under rTMS over the right PFC, participants react less sensitively to their own face if they dislike their own face, compared to participants who like their faces. . The authors speculatively suggested that virtual lesions of the right DLPFC induced by rTMS might reduce self-criticisms in general.

**Heinisch and colleagues (2012)** followed up on their previous study (Heinisch et al., 2011) to further address whether the bias towards the self-induced by rTMS is a result of impaired other recognition or enhanced self-recognition. Uddin et al. (2006) and Heinisch et al. (2011) did not answer this question because the effect of rTMS on other-other discrimination remains unknown. Heinisch et al. (2012) used a task similar to Heinisch and colleagues (2011), with a sham-control condition added for the present study to replace the left TPJ stimulation used by Heinisch et al. (2012). Again, CP6 (midpoint of C6 and P6) was chosen as the site of stimulation to target the right TPJ. The result showed that compared to the sham condition, participants took longer to identify the famous person compared to the self after rTMS stimulation to the right TPJ. Further, no difference in RT was found in the comparison between unfamiliar and famous person identifications. This result supported that deactivating rTMS over the right TPJ enhanced self-recognition and suggested that the right TPJ is not involved in other-other discrimination.

**Payne and Tsakiris (2017)** tested the effects of tDCS vs. sham on the TPJ by stimulating the right temporoparietal area (electrode position: CP6) with a similar video morphing SODT used by Heinisch and colleagues, (2011) but without the unfamiliar face condition. The famous person in this study was gender and skin tone matched with the participant. Participants received either anodal or cathodal 1-mA tDCS over CP6 with the reference electrode placed at the vertex for 20 min. Results indicated that compared to the cathodal or sham condition, anodal tDCS to the right TPJ caused participants to bias towards the other than to the self. This finding suggested that the facilitation of the right TPJ led to inhibited self-recognition. This finding is consistent with previous low-frequency rTMS studies that found TMS inhibition to the right TPJ is associated with enhanced self-recognition (Uddin et al. 2016; Heinisch et al., 2012). The authors suggested that this low-level self-other discrimination in the right TPJ might underly high-level social-cognitive functions like empathy, which involves the inhibition of the self to facilitate the accurate representation of the other.

***Rubber hand illusion (RHI)***

Body ownership, the feeling of being in one's own body, along with movement agency, is one the results of our bodily self-consciousness (Karabanov et al., 2017). The most used experimental paradigm to study the neural basis of body ownership is through the rubber hand illusion (RHI). The procedure typically involves visual occlusion of one arm and substitution of the real occluded arm with a rubber hand within the visual field. Synchronous physical stimulations are then applied to both the real occluded hand and the visible rubber hand, creating the illusion that the rubber hand may be the real hand. The RHI is the temporary shift of body ownership from one's own hand to a rubber hand through multisensory matching. During the matching process, participants will have to focus their attention on the tactile sensations in their hands for the illusion to take place. This focus creates an exteroceptive embodied experience where bodily self-consciousness is effectively engaged. Below we will review studies that implemented NIBS to modulate the effect of the RHI.

**Tsakiris et al. (2008).**Tsakiris and colleagues (2008) used the RHI to study the effects of single-pulse TMS over the right TPJ on NV-SRP. Using a within-subjects design, ten participants (6F/4M) completed the RHI while under real TMS over the right TPJ, the vertex, and in a no TMS condition. TMS intensity varied for each participant to ensure no discomfort occurred, with a mean of 51% stimulator output. Participants were stimulated 350 ms after visual-tactile stimulation applied on the middle finger, followed by a proprioceptive judgement on their middle finger's perceived location. Participants completed the task using their left hand and a rubber hand or their left hand and a spoon as a control. The researchers measured proprioceptive drift, which was the change in the hand's perceived position after TMS compared to the baseline measures taken before the experiment. A positive score of proprioceptive drift is when the participant erroneously localizes his/her hand towards the non-self object (rubber hand or spoon). Typically, without TMS, participants would attribute the rubber hand rather than the spoon to their own body as the result of RHI. This study's results showed that real TMS over the right TPJ was found to reduce proprioceptive drifts significantly when viewing the rubber hand, but increased drifts significantly when viewing the spoon. Essentially, single-pulse TMS over the right TPJ created misattribution of objects during RHI, suggesting the right TPJ's vital role in body ownership. In other words, single-pulse TMS over the right TPJ blurs the boundary between stimuli that belong to one's own body and those that do not belong. One of the limitations of this study is that the researchers used a small sample size (*n* = 10). The role of the right TPJ in non-verbal SRP would be better established by confirming the results of this study with a larger sample size.

**Kammers et al. (2009).** Kammers and colleagues (2009) investigated the effect of rTMS over the left IPL on the RHI. In the first of the two testing sessions, fourteen right-handed participants (9F/5M) received either real or sham rTMS over their left IPL for 20 mins before the RHI. During RHI induction, participants' real right index finger and the rubber hand's index finger were synchronously or asynchronously stroked. After RHI induction, participants immediately indicated the perceived location of their stimulated right index finger and non-stimulated left index finger (as control) and the difference between the actual location and the perceived location was measured as proprioceptive drift. This is measured as the immediate perceptual response. RHI strength was defined as the difference in endpoint error between the synchronous experimental condition and the asynchronous control condition. Subsequently, participants performed four types of actions where they attempted to use the index finger of one hand to reach the index finger of the other hand in four different ways: 1) reaching twice with the non-stimulated left hand; 2) reach once with the stimulated hand and once with the non-stimulated hand; 3) repeat 2) in reversed order; 4) reaching twice with the stimulated hand. This second task was to examine the effect of RHI on proprioceptive drifts over time. After the actions, participants perceived location of their right and left index fingers were measured again as the delayed perceptual response.

First, the researchers confirmed the RHI effect from the immediate perceptual task: increased proprioceptive drift was found after synchronous stroking. Results indicated that rTMS over the left IPL significantly reduced the strength of the illusion only in the synchronous stroking condition. Further, the right hand was slower to reach the target compared to the left hand, suggesting a degree of uncertainty about the starting position of the right hand as the result of RHI. Finally, the results from the delayed perceptual response task suggested no effect of rTMS. After the experiment, participants in the real rTMS group did not report a difference in their subjective perception of the RHI compared to the sham control group. Given the results of this study, the authors suggested that rTMS over the left IPL reduced the RHI for immediate but not for delayed perceptual responses. The authors argued that such effects could not be accounted for by the effect of rTMS on the motor system, nor did the stimulation create changes in subjective experiences of hand ownership.

The authors suggested that this study supported the theoretical model that the IPL is involved in maintaining the spatial configurations of one's body (Dijkerman & de Haan, 2007). Interestingly, it was observed in this study that the attenuated RHI strength was recovered during the second perceptual response. The authors suggested that other systems might be in play to compensate for the effects created by deactivating the IPL. However, future testing is needed for these interpretations.

**Wold et al. (2014).** The extrastriate body area (EBA) has also been implicated in the RHI literature in its role in explicit identity representations and body ownership (Urgesi et al., 2007; Limanowski et al., 2014). To investigate the function of the EBA in body ownership, Wold and colleagues (2014) used rTMS over the EBA to test for its effects in RHI with nineteen participants (11F/8M). Participants' baseline proprioceptive judgments were recorded at the beginning of each session, followed by RHI induction with synchronous and asynchronous strokings. Participants were asked to click a button when they detected RHI onset. After the initial four stroking sessions, participants underwent real or sham 1-Hz rTMS over the left EBA for 20 mins and then repeated the same four stroking sessions. In this experiment, proprioceptive drift was measured by participants' self-reported perceived location of their index finger of their real but invisible right hand. Participants reported their subjective intensity of rubber hand ownership on a 7-point Likert scale after each stroking block.

Results on subjective reports indicated that participants' subjective feelings did not differ due to the stimulation. The authors argued that this might be due to a ceiling effect of the reported illusion, where the rating scales are not sensitive enough to detect changes in the subjective experience. However, even though gender has been implicated as a significant factor in RHI strength, this study did not find an effect of gender on both measured RHI and subjective ratings.

Results on proprioceptive drift indicated that participants experienced increased proprioceptive drift in the synchronous stroking condition following rTMS over the EBA compared to baseline, but not in the sham or asynchronous stroking conditions. This result supports a causal role of left EBA in one’s representation of their own body.

As Tsakiris and Haggard (2005) suggested, proprioceptive drift combines processes from visual, tactile, and proprioceptive systems. Taken together, the authors suggested that the EBA might represent an intermediate level of a hierarchical network of brain areas involved in the RHI. Tsakiris (2010) further suggests that a pre-existing model is representing our own body. During RHI, certain "comparator" (e.g. TPJ) brain areas can match the dummy hand with the model of one's own body, creating a sense of ownership for the rubber hand. The authors suspect that the EBA might also be part of this process of foreign vs. domestic body part comparison, possibly by combining somatosensory and proprioceptive information. Moreover, given the results that the TPJ reduced RHI in the prior study of Tsakiris et al. (2008), the results of this study supported the theoretical model proposed by Arzy et al. (2006) that the EBA might be involved in embodiment processing, which is the sense of being localized within one's physical body. In contrast, the TPJ might be involved in disembodiment processing, which is the sense of being separated from one's physical body. The feeling of embodiment and disembodiment might both contribute to our sense of body ownership.

**della Gatta et al. (2016).** della Gatta and colleagues (2016) investigated the effect of single-pulse TMS over the primary motor cortex (M1) on RHI. Fifty-two right-handed participants (20M/32F) were enrolled in this study half of whom participated in the main experiment (n=26) while the remaining took part in a control experiment (n=26). In the main experiment, participants' MEP was recorded for the stimulated right hand, and the TMS pulse was applied to the corresponding left M1. Participants in the control experiment had their MEP recorded for the unstimulated hand with TMS pulses applied to the corresponding right M1. All participants underwent a standard RHI procedure with synchronous and asynchronous stroking. Proprioceptive drift was measured by participants’ perceived location of their unseen real hand. During one block of RHI induction, participants were first stroked for 12 seconds, then received a single pulse TMS followed by another 12 seconds of stroking in the same condition. After the RHI was measured, participants in the main experiment self-reported their subjective experience of rubber-hand embodiment while participants in the control experiment also reported their disembodiment towards their real hand.

Physiological results indicated that participants' MEP exhibited a significant decrease in the synchronous condition compared to both the asynchronous and the baseline conditions. The analysis of the MEP time course suggests that such an inhibitory effect increased over time. On the contrary, no effect of condition or time was found in the control experiment. Behavioural results indicated that for both the main and the control experiment, proprioceptive drift in the main experiment exhibited a significant increase for synchronous stroking compared to asynchronous stroking, and participants also reported significant increases in the sense of embodiment for synchronous stroking compared to asynchronous stroking. Comparably, in the control experiment, participants reported a decreased feeling of disembodiment for asynchronous stroking compared to synchronous stroking. This study demonstrates that motor movement interacts with our sense of body ownership, contributing to our overall sense of NV-SRP and the feeling of our own body being separated from the external world.

**Karabanov et al. (2017).** Given neuroimaging studies that investigated the role of the ventral premotor cortex (PMv) and the anterior intraparietal sulcus (aIPS) in body ownership, with paired-pulse transcranial magnetic stimulation (ppTMS) this study investigated the effective connectivity between the PMv and the aIPS. More specifically, the researchers used ppTMS as a conditioning pulse over the aIPS and measured a test pulse over the primary motor cortex (M1). Given the test pulse over the M1 can cause a twitch in a target muscle, the conditioning pulse can modulate the aIPS-PMv-M1 pathway (Koch et al., 2010), effectively regulating the effect of the test pulse.

Twenty-eight healthy participants took part in this study (12F/18M, seven participated in the single-pulse experiment 1, and 21 participated in the pair-pulse experiment 2. For the experimental task, participants sat in a chair with their left arm extended on a table. A rubber hand was placed over the participant's hand to occlude participants' vision of their real hand. Participants' index finger was connected to the index finger of the rubber hands via a rod, allowing the index finger of the rubber hand to parallel the movement of the participants' real hand.

The participants were assigned to one of three conditions. In the agency and ownership condition (AO), the rubber hand's position was anatomically plausible (vertically overlap the position of the participants' real hand). In the agency but not ownership condition (ANO), the rubber hand's position was anatomically implausible (positioned in the opposite direction of participants' real hand). Finally, in the no agency and no ownership condition (NANO), the rod was detached, thus, the rubber hand did not mimic the movements of participants' real hand in the NANO condition. In all three conditions, participants were asked to sweep their left index finger sideways at a steady rate of 1 Hz per cycle and participants' electromyogram signals were measured as the primary outcome of the experimental manipulations. Seven participants took part in the single-pulse TMS experiment (experiment 1) targeting the M1 while twenty-one participants participated in the paired-pulse experiment (experiment 2) where an additional coil targeted the right aIPS. The effective connectivity between aIPS and M1 was measured under the three previously described conditions (AO, ANO, NANO) plus a resting condition where participants simply looked at their real hand.

The single-pulse experiment indicated that the MEP did not differ for the AO, ANO, and NANO conditions. Experiment 1 in this context is essential since it rules out that ppTMS modulates the M1 directly instead of modulating the aIPS-M1 connectivity. In terms of the MEP results in the paired-pulse experiment, both resting and the AO condition exhibited negative MEP, whereas the ANO and NANO conditions exhibited positive MEP. Further, the MEP in the resting condition was significantly different from the ANO and NANO condition but not the AO condition. The MEP in the AO condition was also significantly different from the ANO and NANO condition. These results indicated that aIPS serves an inhibitory effect on M1 while processing congruent sensorimotor stimuli (in the rest and AO conditions). Such an inhibitory effect of the aIPS is dampened during sensorimotor conflicts (ANO and NANO conditions).

Additionally, participants experienced increased proprioceptive drift in the AO condition compared to the NAO and NANO conditions. Concerning subjective reports of RHI in the first experiment, participants in the AO and NAO conditions reported an increased sense of agency compared to the NANO condition. Participants in the AO condition also reported an increased sense of ownership compared to the NAO and NANO conditions. In comparison, Experiment 2 did not find significant changes in proprioceptive drifts. The researchers argued that this study was not designed to address the behavioural effects of M1-aIPS stimulation, so these behavioural results should be interpreted cautiously. Another limitation of this study is its a priori hypothesis, and task-unrelated events could cause the results. However, given the inhibitory function of the aIPS during body ownership tasks was also supported by fMRI studies (Shimada et al., 2005; Gentile et al., 2013), the authors argued that task-unrelated events were unlikely the cause for the observed effects.

**Convento et al. (2018).** One model for the RHI suggests an interaction between low-level multisensory integration and high-level incorporation of the rubber hand to one's body. Convento and colleagues (2018) tested the hypothesis that the functional difference between the PMc and the TPJ might reflect this low- and high-level segregation in RHI, respectively. Fifty-six participants (53F/3M) were enrolled in experiment 1, and sixty participants (34F/26M) were enrolled in experiment 2 in a between-subject design. In experiment 1, after a baseline measure of proprioceptive judgement (a procedure similar to della Gatta et al., 2016) of their left index finger, participants received 3 mins of tDCS at rest. Then, participants underwent RHI induction (synchronous or asynchronous) while receiving tDCS for 2 mins, followed by an RHI questionnaire measuring participants' subjective experiences. This tDCS-RHI + questionnaire procedure was then repeated after a one-minute resting period. Therefore, participants received tDCS for the entire experiment except for the initial measurement of their proprioceptive baseline, and their subjective experiences were reported twice at minutes 5 and 10 into the onset of tDCS. In experiment 2, participants merely looked at the rubber hand with no stroking. Since the aim of experiment 2 was only to assess the effects of tDCS on low-level sensory recalibration, no questionnaire was provided in experiment 2. Proprioceptive drift was measured again at the end of both experiments. For tDCS, participants received 1.5 mA anodal stimulation over the right TPJ (node CP6, international 10-20 system) or the right motor area (because the RHI was only done on the left hand) with the cathode placed at the contralateral supraorbital region for both conditions.

Results from experiment 1 indicated that anodal tDCS over both rPMc and right TPJ increased proprioceptive drift to the same degree in both synchronous and asynchronous stroking, suggesting that both areas are involved in proprioceptive localization. Furthermore, anodal stimulation over the right TPJ increased proprioceptive drift in synchronous stroking compared to asynchronous stroking, while the effects of anodal rPMc stimulation on proprioceptive drift were indifferent to synchrony. Additionally, tDCS over the right TPJ induced subjective feelings of "an illusory touch from the rubber hand" as assessed by the questionnaire, which was not seen in the right PMc tDCS. This finding supports the theory that the TPJ is involved in a "test-for-fit" process that compares external stimuli to existing representations of our own body (Tsakiris et al., 2008). Further, results from experiment 2 indicated that anodal stimulation to both the right PMc and right TPJ increased proprioceptive drift even without stroking. The increases in proprioceptive drift induced by rPMc stimulation were also significantly higher than right TPJ stimulation in experiment 2. The finding that rPMc tDCS induced proprioceptive drift but did not induce subjective changes provided support for the r-PMc's involvement in low-level proprioceptive recalibration but not high-level embodiment. As for tDCS's effect in right TPJ without stroking, the authors argued that this effect might be confounded by the anatomical proximity between the TPJ and other RHI-related areas such as the PPC and the IPS. Overall, the results of this study provided support for the existence of at least a dual subcomponent of RHI processing.

**Bassolino et al. (2018).** Bassolino and colleagues (2018) investigated the effect of TMS on the subjective feeling of embodiment during virtual reality (VR) induced RHI. All thirty-two participants (16F/16M) underwent supra-threshold (130% RMT) TMS over their M1 in addition to one of subthreshold (80% RMT at M1) or vertex (130% RMT at M1 vertex, node Cz) control conditions. In the experiment, participants' baseline proprioceptive drift and cortical excitability were first recorded before RHI induction. Then, participants received pulses of TMS to their M1 in short blocks (1 pulse in 10 seconds) during VR-induced RHI. In the synchronous condition, the virtual hand twitched in parallel as participants' real hand twitched, while during the asynchronous condition the virtual hand twitched with increasing times of delay after participants' hand twitched (as caused by the TMS). Participants' proprioceptive drift was measured again after each TMS-VR session. This TMS-VR to proprioceptive drift measurement procedure was then repeated six times, followed by a final session of TMS-VR stimulation. Participants reported their subjective sense of embodiment with questionnaires at the end of the experiment consisting of two questions that belonged to four categories: 1) Ownership of the virtual hand (e.g. "it seemed like the virtual hand was part of my body," p.793); 2) disownership of one's own hand (e.g. "it seemed like my hand had disappeared," p.793); 3) location (e.g. "it seemed like my hand was in the location where my hand was," p. 793); and 4) agency (e.g. "it seemed like I was in control of the virtual hand," p.793). Two additional questions were also included ("it seemed like I had more than two hands") to control for differences in compliance or suggestibility.

Positive proprioceptive drifts towards the virtual hand were found in all conditions. For the main findings, a comparison of the two supra-threshold conditions yielded that only after M1 but not vertex stimulation. participants reported an increased sense of ownership in the synchronous condition compared to the asynchronous condition. No significant effect was found for the other components of the questionnaire. Comparison of the M1 conditions (with different intensities) indicated that only after supra-threshold TMS and not subthreshold TMS participants reported an increased sense of ownership and agency in the synchronous condition when compared to the asynchronous condition. Further, the MEP data suggests that these increases cannot be accounted for by differences in MEP or TMS-evoked hand movements. This study demonstrates that multisensory inputs combined with sensorimotor information were sufficient to produce the sense of body ownership and agency without volition, planning, and anticipation.

**Fossataro et al. (2018).** Fossataro and colleagues (2018) used offline rTMS to investigate the causal role of the M1 in RHI experiences. Thirty-two participants were assigned to either the left-hand or right-hand RHI group after undergoing real or sham rTMS on different days. Participants' MEP was first recorded at the beginning of the experiment, then rTMS was applied over the left M1 for 20 mins to stimulate the right hand. Participants completed the standard RHI procedure with synchronous and asynchronous stroking immediately after rTMS and proprioceptive drift was measured pre- and post-RHI. After the post-RHI proprioceptive drift measure, participants completed a questionnaire assessing their subjective feelings of embodiment of the rubber hand or disembodiment of their real hand. Participants' MEP was recorded again at the end of the experiment.

The analysis first confirmed the inhibitory effects of rTMS on all measures of the RHI (embodiment, disembodiment, proprioceptive drift, and M1 excitability) in both synchronous and asynchronous conditions. Results indicated that only participants in the right-hand RHI condition reported more proprioceptive drift as well as higher ratings of embodiment and disembodiment in the synchronous condition as the results of real rTMS. This result could not be accounted for by differential rTMS M1 inhibition between the left- and right-hand conditions. In other words, rTMS was only effective for the contralateral M1 area during synchronous stroking. The results suggested that down-regulation of the motor system attenuates body ownership, making participants more susceptible to incorporating alien body parts.

**Hornburger et al. (2019).** Hornburger and colleagues (2019) tested the effect of cathodal tDCS over the somatosensory cortex on RHI. Thirty participants (18F/12M) underwent an RHI procedure where the location of the rubber hand shifted to become increasingly anatomically implausible. Participants' baseline proprioceptive drift was measured when their hands were 17.5 cm to the left from their real right hand. Participants then underwent either anodal, cathodal, or sham tDCS over their primary somatosensory cortex (C3 of the 10-20 system) for 20 mins; the reference electrodes for all three conditions were placed in the right supraorbital area. All participants underwent three tDCS protocols on different sessions with counterbalancing. After each 5 minute interval of tDCS, the RHI procedure was repeated 10 cm further from the original hand and the proprioceptive drift and the strength of the illusion at each distance was measured, with participants verbal reports of their right middle finger's position as indicators of their proprioceptive drift. Further, participants were surveyed about specific aspects of the RHI (e.g. "I feel as if the rubber hand was my own hand," p.354) and about the RHI generally (e.g. "My own hand felt artificial," p.354). The experiment concluded after the RHI was examined at the furthest distance (67.5 cm).

Analyses confirmed a stronger RHI effect for synchronous stroking compared to asynchronous stroking. Further, as predicted, RHI strength and proprioceptive drift exhibited gradual decreases as the rubber hand moved further away from the real hand. Results showed that while cathodal tDCS over S1 induced increases in specific aspects of RHI score compared to anodal tDCS, neither of the real stimulation produced significant effects compared to sham. and there were no effects of tDCS on "general" RHI questionnaire scores or proprioceptive drift. The authors suggested that the suppression of the somatosensory cortex by tDCS might play a role in resolving the conflict between visual and proprioceptive input. As the rubber hand positions become increasingly implausible, less regulatory input was needed from the somatosensory cortex. In addition, the null results for proprioceptive drift might indicate a different underlying mechanism for body ownership and proprioception. However, neither of the experimental conditions produced significantly different results from the sham condition, thus the authors argued that future studies should implement a more focal stimulation approach. Finally, since the rubber hand was moved gradually away from the position of the real hand in sequential order, the effects within a session can become confounded. Although this limitation did not affect between-condition comparisons, future studies should investigate the effect of conflict between visual and proprioceptive signals in the RHI with an improved experimental design.

**Lira et al. (2018)** investigated the role of the posterior parietal cortex (PPC) and the ventral premotor cortex (PMv) in the RHI with a large sample. One hundred and sixty right-handed participants (114F/46M) were randomly assigned to one of four groups, resulting in 40 participants per group: 1) synchronous PPC group; 2) asynchronous PPC group; 3) synchronous PMv group; 4) asynchronous PMv group. Each participant underwent three separate sessions of tDCS, receiving one of each tDCS: 1) Anodal on the target area and cathodal on the contralateral supraorbital area; 2) Cathodal on the target area and anodal on the contralateral supraorbital area; 3) Sham stimulation. For PPC and PMv stimulation, the target electrodes were placed at P4 and fC4 according to the 10-10 EEG system. In each session, the strength of tDCS was 1-mA over the PPC and 2-mA for the PMv to achieve similar values of current density in the two areas due to differences in electrode sizes. The order of events during one active tDCS session was: 5 min of tDCS - 30s for the first proprioceptive drift measure - 3 mins of real/sham tDCS with RHI - 30s for the second proprioceptive drift measure. For the proprioceptive measures, participants were asked to locate their right hand using the left index finger. During the 3 mins involving the RHI, participants reported the time of RHI onset verbally. Finally, at the end of the experiment, participants completed a questionnaire that measured the intensity of body ownership (e.g. "It seemed the rubber than was part of my body," p. 2937) and body "control" (e.g. "It felt as if my (real) hand were drifting towards the rubber hand," p.2937).

As predicted, synchronous stroking increased proprioceptive drift compared to the asynchronous condition indifferent to tDCS status. Results indicated that anodal tDCS over the PPC but not the PMv reduced the onset time of RHI regardless of synchrony, compared with the sham control group. Based on this result, the authors suggested that PPC plays a role in multisensory integration during RHI. Interestingly, anodal tDCS over the PPC also affected the asynchronous condition, albeit with a lower magnitude than synchronous stroking. The authors argued that this effect might reflect that our proprioceptive schemas are related to degrees of multisensory integration, which is related to activities in the PPC. Additionally, anodal tDCS over the PPC but not PMv induced significant increases in self-reported body ownership and agency in the synchronous and asynchronous condition, compared to the cathodal and sham conditions. This effect further suggests that anodal tDCS over the PPC can modulate the subjective experiences of the RHI beyond low-level sensory integration.

**Peviani et al. (2018)** investigated the effects of rTMS over the ventral premotor cortex (vPMC) on the RHI based on neuroimaging studies that found greater activation not only in the PPC but also the vPMC during the RHI (Brozzoli, Gentile, & Ehrsson, 2012). Participants (n = 29) were given 1 Hz rTMS at 100% of their motor threshold for 15 minutes over the right VPMC or the vertex as a control, and RHI induction took place before and after stimulation. For proprioceptive drift measures, participants verbally reported their perceived location of the stimulated index finger and completed a body ownership questionnaire after each RHI session. This study's results indicated that rTMS over the vPMC reduced proprioceptive drift without influencing the subjective sense of ownership. This result suggests that the vPMC may play an indirect role in NV-SRP, where stimulation of the vPMC correlates with other brain regions or neural networks that determine the sense of ownership.

***Interoception***

Above we discussed the effect of NIBS on proprioception via the RHI, which involves the processing of exteroceptive signals coming from the hands and lower arm of the outer body (Pollatos et al., 2016). In comparison, below we focus on studies that investigated the effects of NIBS on interoception, involving the processing of signals originating from the inner body. Interoceptive accuracy is considered the core measurement of interoception, usually implemented with a heartbeat counting task (Pollatos & Herbert, 2018). Neuroimaging studies have identified networks of areas including the insula, the ACC, and the somatosensory cortices as the interoceptive neural network (Craig, 2009). NIBS studies can help establish the causal role of each node of the network in interoception.

**Pollatos et al. (2016).**Pollatos and colleagues (2016) investigated the effects of continuous theta-burst stimulation (cTBS) on heartbeat counting accuracy with eighteen healthy male participants. The experiment consists of three sessions where participants received one of three cTBS in each session: 1) right insula (FT8); 2) the somatosensory cortex (Cz); 3) central occipital cortex (Oz). Upon entering the experiment, participants completed an interoceptive sensibility state questionnaire to assess their momentary subjective perception of bodily sensations of their five bodily systems (heartbeat, respiration, trembling, temperature/sweating, and gastrointestinal sensations). After the questionnaire, participants received cTBS over one of the three aforementioned regions, followed by the interoceptive sensibility state questionnaire again for a post-stimulation measurement. Then, participants completed a heartbeat counting task where their accuracy and perceived confidence in the judgment were recorded. Finally, participants completed a respiratory load estimation task where they were instructed to expire maximally with constant flow and asked to indicate their respiratory effort and their confidence in making that judgment. Participants’ EEG data were also collected throughout the experiment to evaluate their interoceptive accuracy in the heartbeat counting task and to assess participants’ heartbeat-evoked potential (HEP), an indicator of cortical response to cardiac interoceptive signals, with or without conscious awareness of cardiac sensations.

The results of this study indicated that: 1) interoceptive accuracy in heartbeat was significantly reduced after cTBS inhibition over the right insula and the right somatosensory cortex, compared to the occipital cortex stimulation; 2) confidence in cardiac interoceptive accuracy was significantly reduced after cTBS inhibition over the right insula compared to the somatosensory cortex inhibition; 3) Insula cTBS induced more anxiety compared to stimulation in the other two locations; 4) interoceptive accuracy in respiration was significantly reduced after cTBS over the right insula compared with the occipital stimulation; 5) confidence in respiratory interoceptive accuracy was significantly reduced after right insula cTBS compared to occipital stimulation; 6) self-reported interoceptive sensibility exhibited significant increase after right insula and somatosensory stimulation compared to before stimulation; 7) participants’ HEP was significantly reduced after cTBS over the right insula, compared to occipital stimulation.

These results supported the right insula and the right somatosensory cortex’s role in interoceptive accuracy, in line with existing findings that the anterior insula is involved in allocating attention to interoceptive signals at the level of conscious awareness. The anterior insula has also been hypothesized to process signals from multiple sources and represent a feeling of uncertainty toward our interoceptive signals. Comparably, the right somatosensory cortex might only be involved in processing interoceptive signals from specific sources.

**Sagliano et al. (2019).**Sagliano and colleagues (2019) also tested the effects of tDCS vs. sham stimulation on the insula with an interoceptive heartbeat counting task with ECG recorded. Before and after tDCS, participants counted their heartbeat at different time intervals and reported their heartbeat count afterwards. For tDCS, anodal stimulation was delivered to the left or right insula (midpoint of F7 and T3 or F8 and T4) and the cathode was placed on the contralateral frontal pole of the stimulation site (Fp1 for right and Fp2 for left). Pre-and post-stimulation results indicated that sham stimulation improved counting accuracy of heartbeats but not real stimulation.

As an interpretation of this seemingly "odd" finding, the authors suggested that real tDCS could have suppressed the "practice" effect seen in the sham condition, which is supported by the finding that participants improved their counting accuracy across conditions. On the neural level, studies have suggested that interoceptive accuracy is associated with functional connectivity between posterior and anterior insula (Keuhn, Mueller, Lohnmann & Schuetz-Bosbach, 2016). The authors also suggested that this result might be due to altered functional connectivity between the posterior and anterior insula caused by tDCS, interfering with the brain activities that promote accurate interoception. In addition to altered functional connectivity within the insula, alterations in top-down attentional control may also underlie the suppressive effect of tDCS in interceptive accuracy. Evidence from a meta-analysis supported this notion and also emphasized the role of the claustrum in deploying top-down attention to interoceptive processing due to its connection with frontal regions (Schulz, 2016). Therefore, Sagliano and colleagues (2019) suggested that their results could be explained by tDCS lowering the allocated attention to the interoceptive task.

**Additional References***

Alon, G., Roys, S. R., Gullapalli, R. P., & Greenspan, J. D. (2011). Non-invasive electrical stimulation of the brain (ESB) modifies the resting-state network connectivity of the primary motor cortex: a proof of concept fMRI study. *Brain research*, *1403*, 37-44.

Anderson, N. H. (1968). Likableness ratings of 555 personality-trait words. Journal of personality and social psychology, 9(3), 272.

Araujo, H. F., Kaplan, J., & Damasio, A. (2013). Cortical midline structures and autobiographical-self processes: an activation-likelihood estimation meta-analysis. Frontiers in human neuroscience, 7, 548.

Arzy, S., Thut, G., Mohr, C., Michel, C. M., & Blanke, O. (2006). Neural basis of embodiment: distinct contributions of temporoparietal junction and extrastriate body area. Journal of Neuroscience, 26(31), 8074-8081.

Axelrod, V., Rees, G., Lavidor, M., & Bar, M. (2015). Increasing propensity to mind-wander with transcranial direct current stimulation. *Proceedings of the National Academy of Sciences*, *112*(11), 3314-3319.

Baeken, C., Remue, J., Vanderhasselt, M. A., Brunoni, A. R., De Witte, S., Duprat, R., ... & Wu, G. R. (2017). Increased left prefrontal brain perfusion after MRI compatible tDCS attenuates momentary ruminative self-referential thoughts. *Brain stimulation*, *10*(6), 1088-1095.

Blanke, O., Ortigue, S., Landis, T., & Seeck, M. (2002). Stimulating illusory own-body perceptions. Nature, 419(6904), 269-270.

Brozzoli, C., Gentile, G., & Ehrsson, H. H. (2012). That's near my hand! Parietal and premotor coding of hand-centered space contributes to localization and self-attribution of the hand. Journal of Neuroscience, 32(42), 14573-14582.

Cabeza, R., & Nyberg, L. (2000). Neural bases of learning and memory: functional neuroimaging evidence. Current opinion in neurology, 13(4), 415-421.

Civai, C., Miniussi, C., & Rumiati, R. I. (2015). Medial prefrontal cortex reacts to unfairness if this damages the self: a tDCS study. *Social Cognitive and Affective Neuroscience*, *10*(8), 1054-1060.

Coulborn, S., Bowman, H., Miall, R. C., & Fernández-Espejo, D. (2020). Effect of tDCS Over the Right Inferior Parietal Lobule on Mind-Wandering Propensity. *Frontiers in Human Neuroscience*, *14*, 230.

Craig, A. D. (2009). Emotional moments across time: a possible neural basis for time perception in the anterior insula. Philosophical Transactions of the Royal Society B: Biological Sciences, 364(1525), 1933-1942.

David, N., Jansen, M., Cohen, M. X., Osswald, K., Molnar-Szakacs, I., Newen, A., ... & Paus, T. (2009). Disturbances of self–other distinction after stimulation of the extrastriate body area in the human brain. *Social Neuroscience*, *4*(1), 40-48.

De Bellis, F., Trojano, L., Errico, D., Grossi, D., & Conson, M. (2017). Whose hand is this? Differential responses of right and left extrastriate body areas to visual images of self and others’ hands. *Cognitive, Affective, & Behavioral Neuroscience*, *17*(4), 826-837.

Denny, B. T., Kober, H., Wager, T. D., & Ochsner, K. N. (2012). A meta-analysis of functional neuroimaging studies of self-and other judgments reveals a spatial gradient for mentalizing in medial prefrontal cortex. *Journal of cognitive Neuroscience*, *24*(8), 1742-1752.

Dijkerman, H. C., & De Haan, E. H. (2007). Somatosensory processes subserving perception and action. Behavioral and brain sciences, 30(2), 189.

Frewen, P., Thornley, E., Rabellino, D., & Lanius, R. (2017). Neuroimaging the traumatized self: fMRI reveals altered response in cortical midline structures and occipital cortex during visual and verbal self-and other-referential processing in women with PTSD. European Journal of Psychotraumatology, 8(1), 1314164.

Gentile, G., Guterstam, A., Brozzoli, C., & Ehrsson, H. H. (2013). Disintegration of multisensory signals from the real hand reduces default limb self-attribution: an fMRI study. Journal of Neuroscience, 33(33), 13350-13366.

Giardina, A., Caltagirone, C., Cipolotti, L., & Oliveri, M. (2012). The role of right and left posterior parietal cortex in the modulation of spatial attentional biases by self and non-self face stimuli. *Social neuroscience*, *7*(4), 359-368.

Guise, K., Kelly, K., Romanowski, J., Vogeley, K., Platek, S. M., Murray, E., & Keenan, J. P. (2007). The anatomical and evolutionary relationship between self-awareness and theory of mind. *Human Nature*, *18*(2), 132-142.

Hooley, J. M., Gruber, S. A., Parker, H. A., Guillaumot, J., Rogowska, J., & Yurgelun-Todd, D. A. (2009). Cortico-limbic response to personally challenging emotional stimuli after complete recovery from depression. *Psychiatry Research: Neuroimaging*, *171*(2), 106-119.

Hoy, K. E., Emonson, M. R., Arnold, S. L., Thomson, R. H., Daskalakis, Z. J., & Fitzgerald, P. B. (2013). Testing the limits: investigating the effect of tDCS dose on working memory enhancement in healthy controls. Neuropsychologia, 51(9), 1777-1784.

Hu, J., Li, Y., Yin, Y., Blue, P. R., Yu, H., & Zhou, X. (2017). How do self-interest and other-need interact in the brain to determine altruistic behavior?. *Neuroimage*, *157*, 598-611.

Kajimura, S., & Nomura, M. (2015). Decreasing propensity to mind-wander with transcranial direct current stimulation. *Neuropsychologia*, *75*, 533-537.

Kajimura, S., Kochiyama, T., Nakai, R., Abe, N., & Nomura, M. (2016). Causal relationship between effective connectivity within the default mode network and mind-wandering regulation and facilitation. *Neuroimage*, *133*, 21-30.

Koch, G., Cercignani, M., Pecchioli, C., Versace, V., Oliveri, M., Caltagirone, C., ... & Bozzali, M. (2010). In vivo definition of parieto-motor connections involved in planning of grasping movements. Neuroimage, 51(1), 300-312.

Krueger, J. (1998). Enhancement bias in descriptions of self and others. Personality and Social Psychology Bulletin, 24(5), 505-516.

Lee, S., Chung, S. W., Rogasch, N. C., Thomson, C. J., Worsley, R. N., Kulkarni, J., ... & Segrave, R. A. (2018). The influence of endogenous estrogen on transcranial direct current stimulation: a preliminary study. European Journal of Neuroscience, 48(4), 2001-2012.

Lee, T. M., Liu, H. L., Tan, L. H., Chan, C. C., Mahankali, S., Feng, C. M., ... & Gao, J. H. (2002). Lie detection by functional magnetic resonance imaging. Human brain mapping, 15(3), 157-164.

LeMoult, J., Kircanski, K., Prasad, G., & Gotlib, I. H. (2017). Negative self-referential processing predicts the recurrence of major depressive episodes. Clinical Psychological Science, 5(1), 174-181.

Liepelt, R., Klempova, B., Dolk, T., Colzato, L. S., Ragert, P., Nitsche, M. A., & Hommel, B. (2016). The medial frontal cortex mediates self-other discrimination in the joint Simon task. *Journal of Psychophysiology*.

Limanowski, J., Lutti, A., & Blankenburg, F. (2014). The extrastriate body area is involved in illusory limb ownership. Neuroimage, 86, 514-524.

Martin, A. K., Dzafic, I., Ramdave, S., & Meinzer, M. (2017). High definition transcranial direct current stimulation over the dorsomedial prefrontal cortex increases the salience of others. *Brain Stimulation: Basic, Translational, and Clinical Research in Neuromodulation*, *10*(2), 422.

McCallion, E., Robinson, C. S., Clark, V. P., & Witkiewitz, K. (2020). Efficacy of Transcranial Direct Current Stimulation-Enhanced Mindfulness-Based Program for Chronic Pain: a Single-Blind Randomized Sham Controlled Pilot Study. *Mindfulness*, *11*(4), 895-904.

Mondino, M., Poulet, E., Suaud-Chagny, M. F., & Brunelin, J. (2016). Anodal tDCS targeting the left temporo-parietal junction disrupts verbal reality-monitoring. *Neuropsychologia*, *89*, 478-484.

Paulhus, D. L. (1998). Interpersonal and intrapsychic adaptiveness of trait self-enhancement: A mixed blessing?. Journal of personality and social psychology, 74(5), 1197.

Pisoni, A., Lauro, L. J. R., Vergallito, A., Maddaluno, O., & Bolognini, N. (2018). Cortical dynamics underpinning the self-other distinction of touch: a TMS-EEG study. *NeuroImage*, *178*, 475-484.

Pollatos, O., & Herbert, B. M. (2018). Interoception: Definitions, dimensions, neural substrates. In Embodiment in Psychotherapy (pp. 15-27). Springer, Cham.

Preston, C., & Newport, R. (2008). Misattribution of movement agency following right parietal TMS. *Social cognitive and affective neuroscience*, *3*(1), 26-32.

Salehinejad, M. A., Nejati, V., & Nitsche, M. A. (2020). Neurocognitive correlates of self-esteem: from self-related attentional bias to involvement of the ventromedial prefrontal cortex. *Neuroscience research*, *161*, 33-43.

Salerno, S., Zamagni, E., Urquizar, C., Salemme, R., Farnè, A., & Frassinetti, F. (2012). Increases of corticospinal excitability in self‐related processing. *European Journal of Neuroscience*, *36*(5), 2716-2721.

Schulz, S. M. (2016). Neural correlates of heart-focused interoception: a functional magnetic resonance imaging meta-analysis. Philosophical Transactions of the Royal Society B: Biological Sciences, 371(1708), 20160018.

Schuwerk, T., Schecklmann, M., Langguth, B., Döhnel, K., Sodian, B., & Sommer, M. (2014). Inhibiting the posterior medial prefrontal cortex by rTMS decreases the discrepancy between self and other in theory of mind reasoning. *Behavioural brain research*, *274*, 312-318.

Shimada, S., Hiraki, K., & Oda, I. (2005). The parietal role in the sense of self-ownership with temporal discrepancy between visual and proprioceptive feedbacks. Neuroimage, 24(4), 1225-1232.

Tsakiris, M. (2010). My body in the brain: a neurocognitive model of body-ownership. Neuropsychologia, 48(3), 703-712.

Tsakiris, M., & Haggard, P. (2005). The rubber hand illusion revisited: visuotactile integration and self-attribution. Journal of Experimental Psychology: Human Perception and Performance, 31(1), 80.

Uddin, L. Q., Kaplan, J. T., Molnar-Szakacs, I., Zaidel, E., & Iacoboni, M. (2005). Self-face recognition activates a frontoparietal “mirror” network in the right hemisphere: an event-related fMRI study. Neuroimage, 25(3), 926-935.

Urgesi, C., Candidi, M., Ionta, S., & Aglioti, S. M. (2007). Representation of body identity and body actions in extrastriate body area and ventral premotor cortex. Nature neuroscience, 10(1), 30-31.

van der Werf, Y. D., Sanz-Arigita, E. J., Menning, S., & van den Heuvel, O. A. (2010). Modulating spontaneous brain activity using repetitive transcranial magnetic stimulation. *BMC neuroscience*, *11*(1), 1-6.

Wagner, D. D., Haxby, J. V., & Heatherton, T. F. (2012). The representation of self and person knowledge in the medial prefrontal cortex. *Wiley Interdisciplinary Reviews: Cognitive Science*, *3*(4), 451-470.

Zhang, Y., Chen, S., Hu, X., & Mai, X. (2019). Increasing the difference in decision making for oneself and for others by stimulating the right temporoparietal junction. *Frontiers in psychology*, *10*, 185.
